# Histone H3K27 demethylation drives Crohn’s disease inflammation: GSK-J4 as a potential epigenetic therapy

**DOI:** 10.1186/s13148-026-02165-2

**Published:** 2026-05-21

**Authors:** Jigisha A. Patel, Kevin Beatson, Jeries Abu-Hanna, Rijan Gurung, Paul Harrow, Christopher Wood, Marilena Loizidou, Lucie H. Clapp, C. Richard Cohen, Mohammad Mahmoud Rajab Eddama

**Affiliations:** 1 Division of Surgery and Interventional Science, Research Department of Surgical Biotechnology, University College London, GI Services, Ground Floor, 250 Euston Road, NW1 2PG London, UK; 2 Gastrointestinal Services, University College London Hospitals NHS Foundation Trust, London, UK; 3 Department of Colorectal Surgery, University College London Hospital NHS Foundation Trust, London, UK; 4 Cardiovascular Sciences, University College London, London, UK; 5 Division of Surgery and Interventional Science, Research Department of Targeted Intervention, University College London, London, UK; 6 Digestive Disease Institute, Cleveland Clinic London, London, UK

**Keywords:** Crohn’s disease, Histone demethylation, H3K27me3, KDM6A/B, GSK-J4

## Abstract

**Background:**

Crohn’s disease (CD) is characterised by recurrent gastrointestinal inflammation. Epigenetic modifications, particularly the methylation of histone H3 at lysine 27 trimethylation (H3K27me3), are crucial in regulating gene expression and macrophage activity. Dysregulation of these modifications may contribute to CD pathogenesis.

**Objective:**

To investigate the role of H3K27me3 in CD, assess the involvement of the WNK1-TAK1 signalling pathway, and evaluate the therapeutic potential of GSK-J4, a selective histone demethylase inhibitor.

**Methods:**

Peripheral blood and intestinal tissue were collected from CD patients (*n* = 19) undergoing surgical resection and from age-matched healthy controls (HC, *n* = 17). Monocytes were isolated and differentiated into macrophages. Expression of H3K27me3, demethylases KDM6A and KDM6B, and components of the WNK1-TAK1 pathway were assessed by Immunofluorescence, Western blotting, and qRT-PCR. GSK-J4 was used to assess its effect on these markers and cytokine secretion.

**Results:**

Inflamed CD tissue and derived macrophages showed increased KDM6A/B expression and reduced H3K27me3 compared with unaffected tissue and HC-derived macrophages. GSK-J4 treatment reversed this epigenetic profile and significantly reduced secretion of inflammatory cytokines IL-6, IL-10, and TNF-α. Activation of the WNK1-TAK1 axis was identified as a key mediator of lipopolysaccharide-induced cytokine release and was modulated by GSK-J4.

**Conclusion:**

This study identifies KDM6A/B-driven epigenetic dysregulation as a novel pathogenetic mechanism in CD. GSK-J4 effectively restores H3K27me3 levels and reduces inflammation, supporting histone demethylase inhibition as a promising and mechanistically novel therapeutic approach warranting further translational investigation.

**Supplementary Information:**

The online version contains supplementary material available at 10.1186/s13148-026-02165-2.

## Background

Crohn’s disease (CD) is a complex, chronic inflammatory bowel disorder that presents significant challenges not only for patients but also for their families and healthcare systems [1]. With a rising prevalence, coupled with a peak incidence in young individuals aged 30 to 40 years, CD imposes a substantial socioeconomic burden, significantly impairing the quality of life for those affected [2, 3]. Current treatment strategies are largely dominated by immunosuppressive therapies and monoclonal antibodies, aimed at introducing remission and preventing relapse. However, response rates are suboptimal, with up to 50% of patients not responding to treatment, and up to 80% failing to achieve remission, reflecting the disorder’s heterogeneity and complications surrounding development of drug resistance [4]. For instance, primary non-response to anti-tumour necrosis factor alpha (TNFα) therapies such as infliximab and adalimumab, is as high as 24% at week 14, while the cumulative non-remission rate escalates to 63% by week 54 [5, 6]. Consequently, surgical intervention becomes a necessary recourse for many patients, emphasising an urgent need for development of target therapies that can sustain long-term remission and reduce the necessity for invasive procedures [7].

In light of the treatment failures and the complexities of surgery, the role of epigenetic changes in the pathogenesis of CD has emerged as a promising avenue for exploration. One key player in this context is the methylation status of histone 3 lysine 27 (H3K27), a critical epigenetic regulator of cellular homeostasis and development [8]. Notably, demethylation of trimethyl H3K27 in response to inflammatory stimuli results in the upregulation of pro-inflammatory cytokines, including interleukin 6 (IL-6), and TNF-α [9–11]. Critical to this process are the enzymes jumonji domain-containing protein-3 (JMJD3), also known as lysine-specific demethylase 6B (KDM6B) and ubiquitously transcribed tetratricopeptide repeat, X-chromosome (UTX), also known as KDM6A, both of which orchestrate the demethylation of H3K27me3. GSK-J4, a recently developed pro-drug, inhibits KDM6A and KDM6B, preserving H3K27 in a trimethylation state and suppressing the transcription of inflammatory cytokines [12]. This pharmacological approach thus presents a promising avenue for modulating inflammatory responses in CD.

While the immunopathogenesis of CD is well characterised by dysregulated innate and adoptive immune responses, including impaired sensing of luminal antigens, altered autophagy, epithelial barrier dysfunction, and sustained activation of NF-κB and MAPK signalling pathway, there is currently no established role for the with-no-lysine kinase 1 (WNK1) pathway in CD pathogenesis [13–15]. Large-scale genome-wide association studies and contemporary mechanistic reviews of CD identify pathways involving NOD2, autophagy, endoplasmic reticulum stress, and cytokine signalling as central to disease susceptibility and progression, but do not implicate WNK1 as a disease-associated locus or canonical inflammatory pathway [13, 16].

Nevertheless, emerging experimental evidence has identified WNK1 as an important negative regulator of Lipopolysaccharide (LPS)-induced inflammatory signalling in macrophages, acting upstream of TGF-β–activated kinase 1 (TAK1) to suppress NF-κB nuclear translocation and MAPK phosphorylation [17]. In macrophage models, genetic or functional loss of WNK1 results in exaggerated TAK1 activation, enhanced NF-κB/MAPK signalling, and increased pro-inflammatory cytokine production following LPS stimulation, whereas WNK1 activation attenuates these responses [17]. Although these findings establish a regulatory role for WNK1 in innate immune signalling, its relevance to intestinal inflammation and CD has not been investigated, and no studies to date have examined modulation of WNK1 pathway in established preclinical models of inflammatory bowel disease (IBD) [18].

**Fig. 1:**
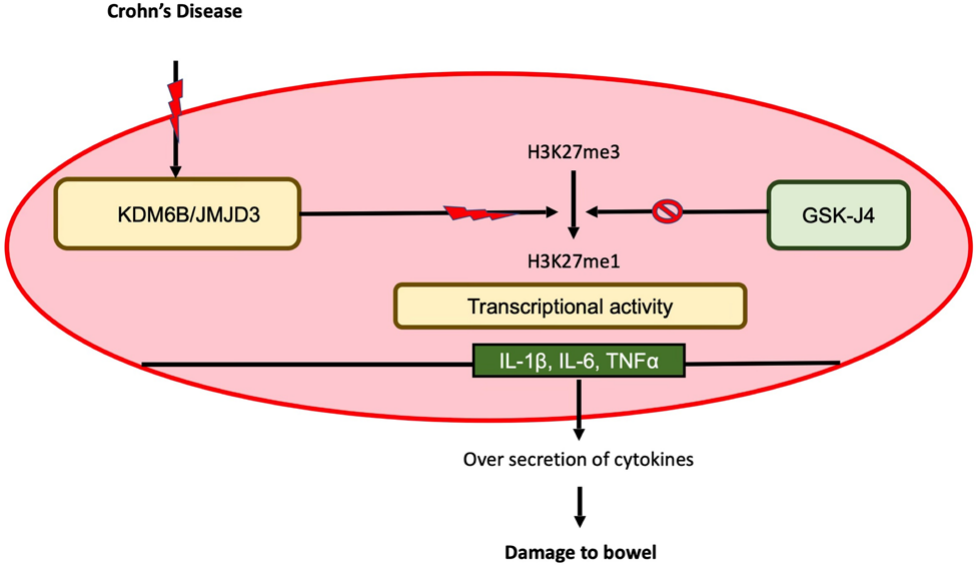
Proposed research hypothesis illustrating that the histone demethylases KDM6A and KDM6B are upregulated in Crohn’s disease macrophages, leading to transcriptional activation of inflammatory cytokines and driving disease pathogenesis. The demethylase inhibitor GSK-J4 is hypothesised to attenuate inflammation by selectively inhibiting KDM6A/B activity

In this study, we hypothesised that dysregulation of WNK1 signalling in CD macrophages permits excessive activation of TAK1, leading to sustained NF-κB and MAPK signalling and amplification of inflammatory responses. We further postulated that this inflammatory signalling environment drives upregulation of histone demethylases KDM6A and KDM6B, resulting in loss of the repressive H3K27me3 mark, thereby contributing to dysregulated cytokine production in CD. Accordingly, we hypothesised that the pharmacological inhibition of KDM6A/B using the selective demethylase inhibitor GSK-J4 could restore H3K27me3 and modulate inflammatory responses in CD macrophages (Fig. 1). Furthermore, our aim was to investigate the potential interactions between H3K27 methylation and the WNK1 signalling pathway, which may contribute to our understanding of the molecular mechanisms underlying CD, identify therapeutic targets, and open avenues for novel epigenetic-based therapeutic interventions.

## Methods

All participants, including patients with CD and healthy controls (HC), were recruited from University College London Hospital (UCLH), London. All CD patients had a confirmed clinical diagnosis based on established endoscopic and histological criteria. Adults (≥ 18 years) undergoing surgical resection for CD were eligible if they could provide written informed consent and had no evidence of acute illness. Patients with other autoimmune disorders, active malignancies, or acute admissions were excluded. HC participants were age-matched volunteers recruited from UCLH and University College London (UCL) staff. This study received ethical approval from the Health Research Authority and Health and Care Research Wales (REC reference 21/NW/0023 and UCL-RFH). All participants provided written informed consent prior to their participation in the study.

Paired macroscopically affected and unaffected tissue samples were collected from surgical resections, with 6 patients (32%) presenting with ileal disease, 6 (32%) with colonic disease and 7 (37%) with ileocolonic disease. Peripheral blood was obtained from CD patients (*n* = 19) and HC (*n* = 17) using lavender ethylenediaminetetraacetic acid (EDTA) vacutainer tubes (BD, Oxford, UK). Monocytes were isolated and differentiated into macrophages for in vitro studies. Baseline demographic and clinical characteristics of participants are detailed in online supplementary Table S1.

Monocytes were isolated from peripheral blood and cultured in the presence of macrophage colony-stimulating factor (M-CSF) for 7–10 days to generate differentiated macrophages. M-CSF differentiation generates unpolarised (M0) macrophages. These cells were subsequently stimulated with lipopolysaccharide (LPS) to induce a pro-inflammatory phenotype and are referred to in this study as LPS-stimulated or M1-like macrophages. Cells were then treated with Foetal Bovine Serum (FBS) only; FBS + LPS; FBS + LPS + 30 µM GSK-J4; and FBS + 30 µM GSK-J4 for 24 h. Cytokine secretion (IL-6, IL-10,TNF-α) was quantified by ELISA. Expression of KDM6A, KDM6B, and H3K27me3 was assessed by immunofluorescence, Western blotting, and qRT-PCR. Cell viability and cytotoxicity of GSK-J4 were measured using an MTS assay. Detailed protocols for tissue immunofluorescence, immunocytology, Western blotting, qRT-PCR, and cell assays are provided in the online supplemental materials and methods section, including antibody information (Table S2).

Statistical analysis was performed with GraphPad Prism (GraphPad Prism v9.1.1.225 for Windows, GraphPad Software, San Diego, CA, www.graphpad.com). Data was compared by one-way ANOVA or Student’s t-test. Results are expressed as mean ± SEM, with *p* ≤ 0.05 considered statistically significant.

### Patient and public involvement (PPI)

Patients and members of the public were involved in all stages of this study, including co-developing the research concept, reviewing the protocol and participant materials, and advising on recruitment and dissemination. A full account PPI contribution is detailed in the online supplementary material (Supplementary method section).

## Results

### The expression of demethylases and H3K27me3 in macroscopically affected CD tissue

**Fig. 2:**
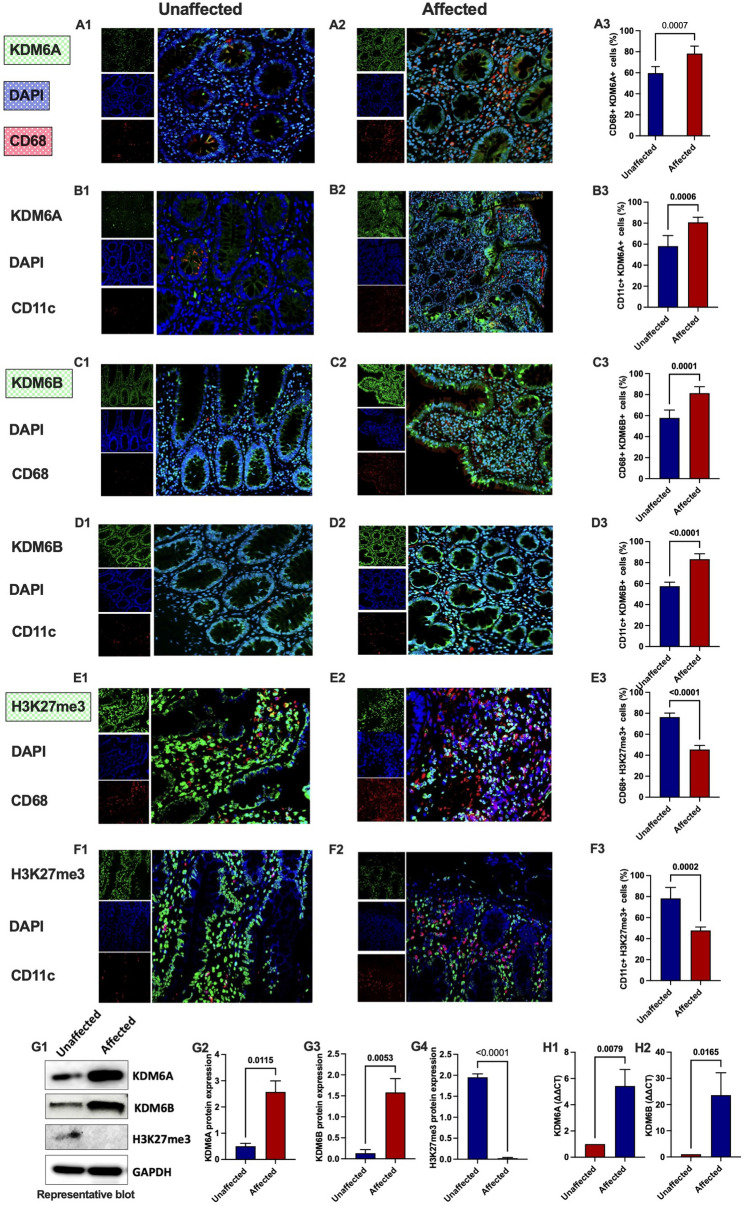
Tissue expression of the histone demethylases KDM6A and KDM6B, and trimethylated histone H3 lysine (H3K27me3), in macrophages within macroscopically unaffected and affected intestinal tissue from patients with Crohn’s disease (CD) (*n* = 7). Immunofluorescence staining of KDM6A (**A1**-**A3**, **B1**-**B3**), KDM6B(**C1**-**C3**, **D1**-**D3**) and H3K27me3 (**E1**-**E3**, **F1**-**F3**) was performed on paired CD-unaffected and CD-affected intestinal tissues sections. Nuclear localisation was quantified using DAPI (blue), and macrophages were identified by co-localisation with the pan-macrophage marker CD68 or pro-inflammatory macrophage marker CD11c (red), with target protein shown in green. Quantification was first performed relative to CD68 macrophages to assess global macrophage expression and subsequently within CD11c macrophage subset to control for macrophage polarisation. Bar graphs represent the percentage of marker-positive macrophages in unaffected versus affected tissue. Representative Western Blot images demonstrate protein expression of KDM6A, KDM6B, and H3K27me3 in paired unaffected and affected tissue samples from CD patients (**G1**), with corresponding densitometric quantification normalised to GAPDH (**G2**-**G4**). qRT-PCR analysis shows mRNA expression of KDM6A (**H1**) and KDM6B (**H2**), with ∆Ct values calculated as absolute expression normalised to β-actin and ∆∆Ct values expressed as fold change relative to the basal condition. Data are shown as mean ± SEM

Immunofluorescence staining of paraffin-embedded colonic tissue demonstrated a significant upregulation of KDM6A (*p* < 0.001) in CD68^+^ macrophages (a pan-macrophage marker) and CD11c^+^ macrophages (a marker of activated/pro-inflammatory macrophages) within inflamed tissue, compared to adjacent non-inflamed tissue from the same patient (Fig. 2A1-A3 and B1-B3). Similarly, KDM6B expression was significantly elevated (*p* < 0.001) in inflamed tissue (Fig. 2C1-C3 and D1-D3).

The increased expression of KDM6A and KDM6B was accompanied by a notable increase in macrophage infiltration within the inflamed colonic tissue compared to unaffected regions. This was associated with a significant reduction in H3K27me3 expression (*p* < 0.001), with approximately a 40% decrease observed in affected tissue (Fig. 2E1-E3 and F1-F3).

Western blotting analysis of lysed colonic tissue confirmed a significant upregulation of both KDM6A (*p* < 0.05) and KDM6B (*p* < 0.01) in affected samples, accompanied by a marked reduction in H3K27me3 protein expression (*p* < 0.001) (Fig. 2G1-G4). Furthermore, qRT-PCR analysis revealed a significant increase in mRNA gene expression of KDM6A (*p* < 0.01) and KDM6B (*p* < 0.05) in affected CD tissue (Fig. 2H1-H2).

### Upregulation of demethylases and downregulation of H3K27me3 in CD-derived macrophages

**Fig. 3:**
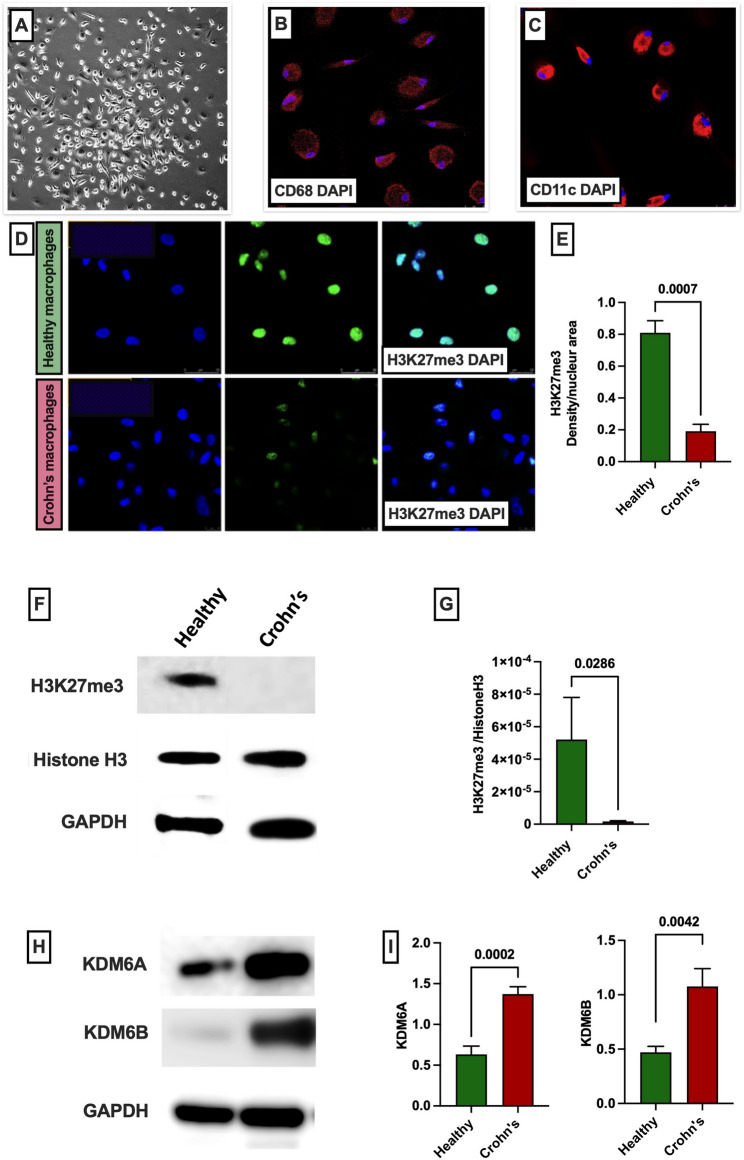
Reduced H3K27me3 and increased KDM6A/B expression in Crohn’s disease (CD)-derived macrophages. Monocyte-derived macrophages were generated from peripheral blood monocytes obtained from healthy controls and patients with Crohn’s disease (CD). Phase-contrast imaging illustrates macrophages morphology following differentiation (**A**), and immunofluorescence staining confirming macrophage identity using CD68 (red) and the pro-inflammatory macrophage marker CD11c (red), with nuclear counterstaining using DAPI (blue) (**B**-**C**). Immunofluorescence analysis demonstrates reduced nuclear expression of H3K27me3 (green) in macrophages derived from CD patients compared with healthy controls, with nuclear localisation defined by DAPI (blue) (**D**). Quantification of H3K27me3 signal intensity normalised to nuclear area is shown in (**E**). Representative Western blot analysis demonstrates reduced H3K27me3 and GAPDH used as loading controls (**F**). Correspond densitometric quantification normalised to Histone H3 is shown in (**G**). In parallel, Western blot analysis demonstrates increase expression of the histone demethylases KDM6A and KDM6B in CD-derived macrophages (**H**) with densitometric quantification normalised to GAPDH shown in (**IA**). Data are presented as mean ± SEM from healthy controls (*n* = 6); and CD patients (*n* = 7). Statistical significance was determined using appropriate two-group comparison; *p* ≤ 0.05 was considered significant

Monocytes from CD patients were differentiated into unpolarised (M0)macrophages using M-CSF and then cultured for 7–10 days until they reached approximately 80% confluency (Fig. 3A). Characterisation by immunofluorescence staining confirmed macrophage identity, with cells staining positive for CD68 (Fig. 3B) and CD11c (Fig. 3C).

Immunofluorescence staining of CD-derived macrophages revealed a significant reduction in nuclear H3K27me3 expression compared to macrophages isolated from healthy controls (Fig. 3D). Quantification using ImageJ showed a significantly lower H3K27me3 density per nuclear area, as determined using the nuclear stain, DAPI (*p* < 0.001) (Fig. 3E).

Western blot analysis of lysed macrophages further confirmed a significant reduction in H3K27me3 protein expression in CD-derived macrophages, which was normalised to total histone H3 and further adjusted using a GAPDH loading control (*p* < 0.05) (Fig. 3: F and G). In the same lysates, KDM6A expression was markedly increased (*p* < 0.001), along with a significant upregulation of KDM6B (*p* < 0.01) in CD-derived macrophages compared to controls (Fig. 3H and I).

### GSK-J4 effect on dysregulated demethylase enzymes and H3K27me3 expression in CD-derived macrophages

To determine whether pharmacological inhibition of KDM6A and KDM6B could reverse the dysregulated epigenetic profile observed in CD-derived macrophages, we assessed the effect of GSK-J4 treatment following LPS stimulation. Primary macrophages were cultured from monocytes from CD patients and treated with FBS-containing growth media, either with or without LPS, LPS + GSK-J4 or GSK-J4 alone for 24 h.

**Fig. 4:**
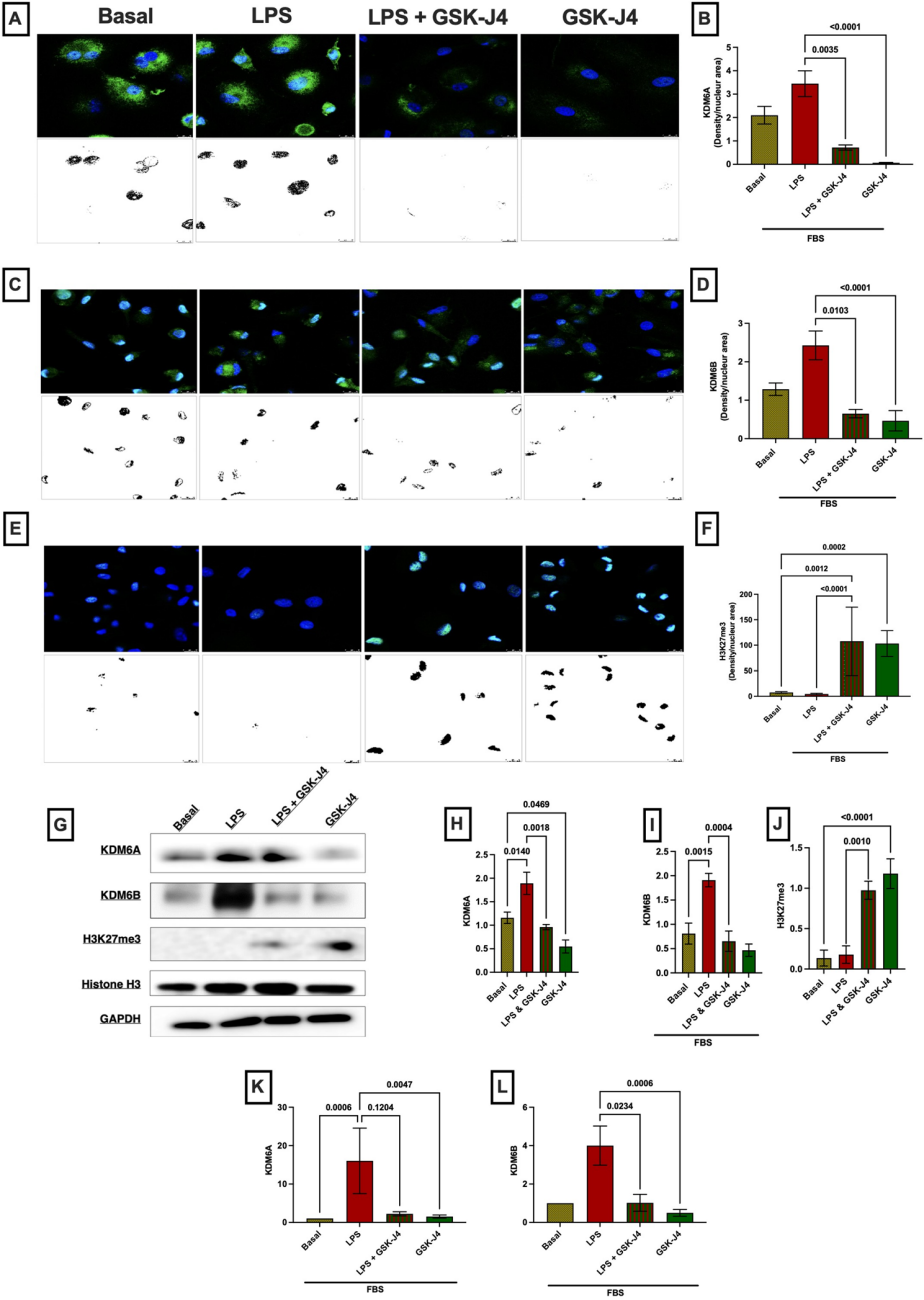
GSK-J4 attenuates dysregulated KDM6A and KDM6B and restores H3K27 trimethylation in Crohn’s disease (CD)-derived macrophages. Monocytes-derived macrophages from patients with CD were cultured under basal conditions (FBS), stimulated with lipopolysaccharide (LPS), or treated with the selective histone demethylase inhibitor GSK-J4 (30 µM) in the presence or absence of LPS for 24 h. Immunofluorescence staining demonstrates changes in nuclear expression of KDM6A (**A**-**B**), KDM6B (**C**-**D**), and H3K27me3 (**E**-**F**), with representative binary images shown beneath each condition. Quantification is presented as nuclear signal density normalised to nuclear area. Representative Western blot analysis shows protein expression level of KDM6A, KDM6B, and H3K27me3 under the indicated treatment conditions (**G**), with histone H3 and GAPDH used as loading controls. Corresponding densitometric quantification normalised to the appropriate loading control is shown in panels (**H**-**J**). Quantitative RT-PCR analysis demonstrates mRNA expression of KDM6A (**KA**) and KDM6B (**L**), with ∆Ct calculated as absolute expression normalised to GAPDH and ∆∆Ct values expressed as fold change relative to the basal conditions. Data are presented as mean ± SEM from macrophages derived from patients with CD (*n* = 8) statistical significance was determined using appropriate multi-group; *p* ≤ 0.05 was considered significant

CD-derived macrophages treated with LPS showed a significant increase in KDM6A and KDM6B expression compared to basal controls. Furthermore, treatment with combined LPS + GSK-J4 significantly reduced the expression of KDM6A and KDM6B (*p* < 0.01 and *p* < 0.05, respectively), with expression level trending towards or below the FBS-only baseline (Fig. 4A-D). Treatment with GSK-J4 alone resulted in further reduction in KDM6A and KDM6B expression, yet more significant (*p* < 0.0001) compared to FBS + LPS treatment (Fig. 4A-D).

On the other hand, H3K27me3 expression exhibited an inverse pattern. LPS-stimulated CD-derived macrophages showed reduced levels of H3K27me3, whereas treatment with LPS + GSK-J4 significantly increased H3K27me3 expression (*p* < 0.0001), a similar effect observed with GSK-J4 alone (*p* < 0.001) (Fig. 4E-F).

Western blot analysis confirmed these findings, showing elevated KDM6A expression in LPS-stimulated macrophages (*p* < 0.05), which was reversed with LPS + GSK-J4 treatment (*p* < 0.01) (Fig. 4G-I). GSK-J4 alone reduced KDM6A expression below baseline (*p* < 0.05). KDM6B expression followed the same trend, increasing with LPS treatment (*p* < 0.01), and reducing with LPS + GSK-J4 (*p* < 0.001). Conversely, H3K27me3 levels were reduced following LPS treatment but were significantly increased following LPS + GSK-J4 treatment (*p* < 0.001) or when treated with GSK-J4 alone (*p* < 0.0001) (Fig. 4G and J).

qRT-PCR analysis mirrored the above protein expression patterns. LPS treatment significantly increased KDM6A mRNA expression compared to FBS-only basal controls (*p* < 0.001), with KDM6B expression showing a similar trend. KDM6A mRNA levels showed a trend towards a baseline following LPS + GSK-J4 treatment, while KDM6B mRNA levels were significantly reduced compared to LPS treatment (*p* < 0.05) (Fig. 4K and L).

Together, these findings demonstrate that GSK-J4 attenuates the dysregulated expression of KDM6A, KDM6B, and H3K27me3 in CD-derived macrophages.

### GSK-J4 effect on the release of inflammatory cytokines, IL-6, IL-10 and TNF-α

IL-6, IL-10 and TNF-α levels were measured in the cell culture supernatant of HC-derived and CD-derived macrophages following treatment with LPS, GSK-J4 + LPS or GSK-J4 only. A time-course experiment of cytokine release in HC-derived macrophages (1, 6, and 24 h) identified progressive cytokine accumulation with peak secretion observed at 24 h, which was subsequently used as the standard treatment duration for CD-derived macrophages and all further experiments.

**Fig. 5:**
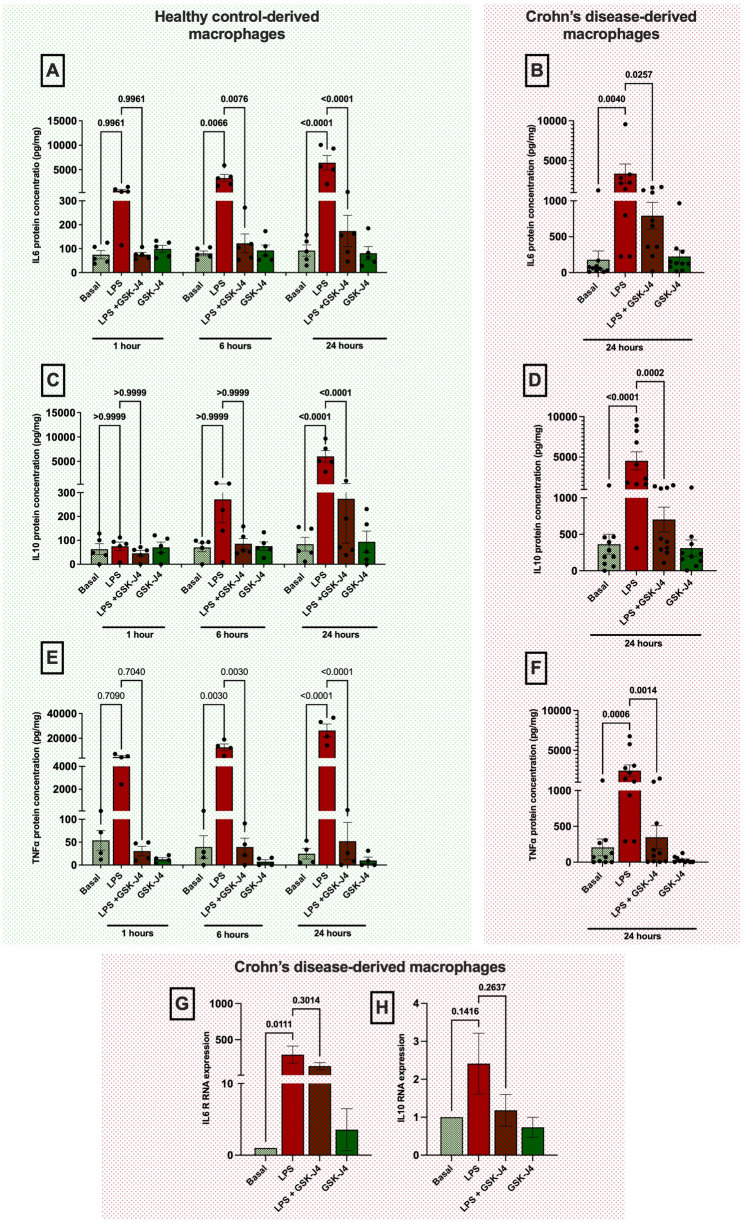
GSK-J4 attenuates Lipopolysaccharide-induced cytokine production in healthy control- and Crohn’s disease-derived macrophages. Monocyte-derived macrophages from healthy controls and patients with Crohn’s disease (CD) were cultured under basal conditions (FBS), stimulated with lipopolysaccharide (LPS), or treated with the elective histone demethylase inhibitor GSK-J4 (30µM) in the presence or absence of LPS. Cytokine concentrations in culture supernatants were measured by ELISA. In macrophages derived from healthy controls, IL6 (**A**), IL10 (**C**), and TNF-α (**E**) production was assessed at 1 h, 6 h and 24 h following treatment. In macrophages derived from patients with CD, cytokine production was assessed at 24 h (IL6: **B**; IL10: **D**; TNF-α: **F**). LPS stimulated significantly increased cytokine production in both healthy control- and CD-derived macrophages, with CD macrophages demonstrating a robust inflammatory response at 24 h. Co-treatment with GSK-J4 attenuated LPS-induced cytokine secretion. Gene expression analysis performed in CD-derived macrophages only demonstrated modulation of IL6 receptor (IL6R) (**G**) and IL10 (**H**) mRNA levels following treatment. ∆Ct values were calculated as expression normalised to GAPDH, and ∆∆Ct values are expressed as fold change relative to basal conditions. Data are presented as mean ± SEM from Healthy controls (*n* = 5) and patients with CD (*n* = 8). Statistical significance was determined using appropriate multi-group comparisons; *p* ≤ 0.05 was considered significant

IL-6 levels were significantly elevated in both HC-derived (*p* < 0.0001) and CD-derived macrophages (*p* < 0.01) treated with LPS compared to basal (FBS without LPS). This increase was significantly attenuated following LPS + GSK-J4 treatment in both HC-derived (*p* < 0.0001) and CD-derived macrophages (*p* < 0.05) (Fig. 5A and B). Notably, HC-derived macrophages demonstrated a greater magnitude of LPS-induced cytokine release compared to CD-derived macrophages.

Similarly, IL-10 release was markedly increased in LPS-stimulated macrophages compared to baseline (*p* < 0.0001). Treatment with LPS + GSK-J4 significantly attenuated IL-10 secretion in macrophages derived from both healthy controls (*p* < 0.0001) and CD patients (*p* < 0.001) (Fig. 5C and D). This increase in IL-10 at 24 h is consistent with its role as a regulatory cytokine produced in response to sustained inflammatory stimulation. TNF-α levels followed the same pattern, showing a significant increase in LPS-stimulated HC-derived (*p* < 0.0001) and CD-derived macrophages (*p* < 0.001) compared to baseline. This response was attenuated in LPS + GSK-J4-treated macrophages, with a significant reduction observed in both HC-derived (*p* < 0.0001) and CD-derived macrophages (*p* < 0.01) (Fig. 5E and F).

The mRNA expression of IL-6 receptor (IL-6R) and IL-10 was assessed by qRT-PCR. IL-6 protein levels reflect cytokine secretion, whereas IL-6R expression was assessed to evaluate potential changes in macrophage responsiveness to IL-6 signalling. While IL-6R mRNA expression was elevated in LPS-stimulated macrophages compared to baseline (*p* < 0.05), LPS + GSK-J4 treatment did not reduce IL-6R expression. Although IL-10 mRNA showed an upward trend in LPS-stimulated macrophages, followed by a return to baseline in LPS + GSK-J4-treated cells, this did not reach statistical significance (Fig. 5G and H).

### Induction of inflammatory cytokines by LPS potentially regulated through WNK1-TAK1 signalling pathway

**Fig. 6:**
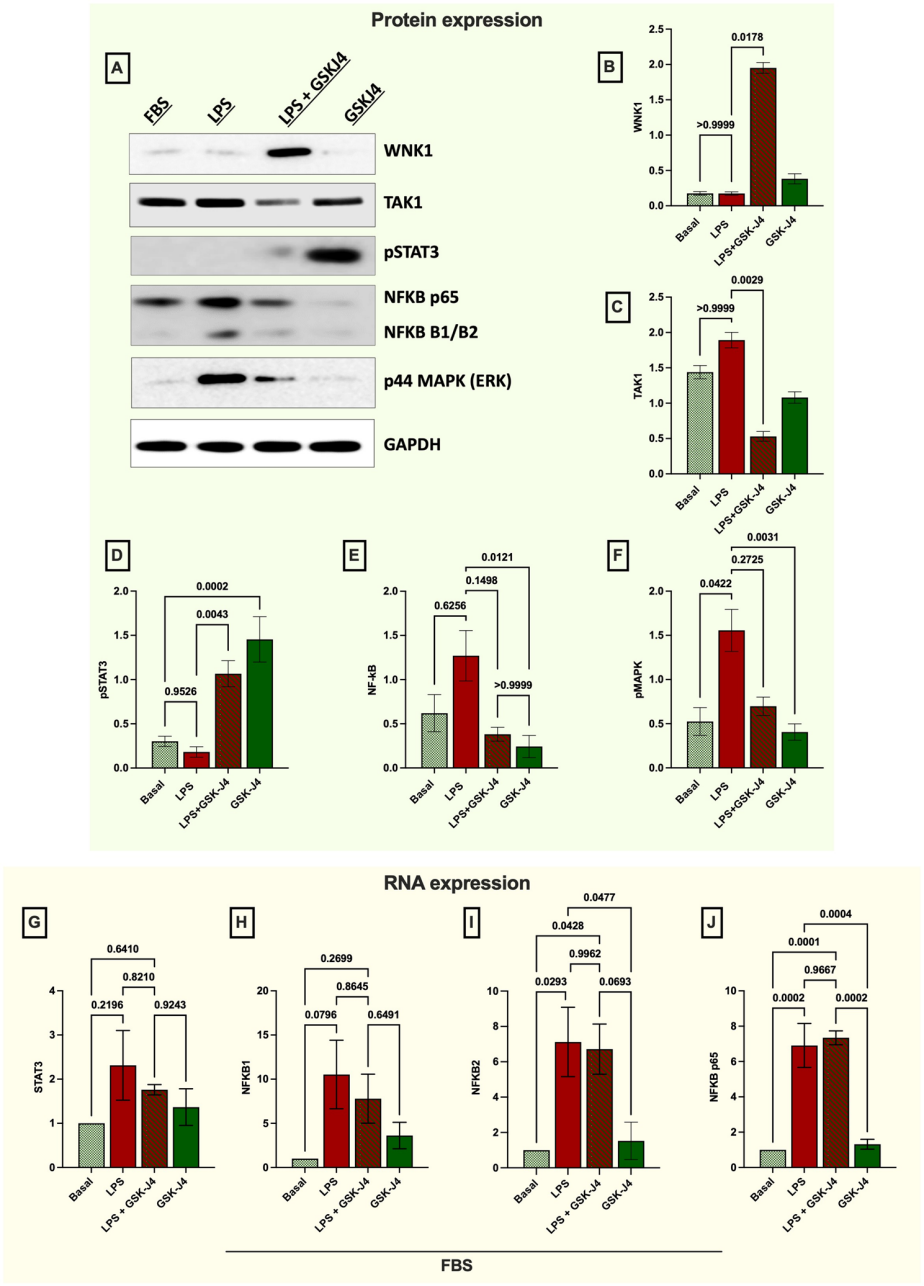
GSK-J4 modulated WNK1-TAK1 signalling axis and downstream inflammatory pathways in Crohn’s disease (CD)-derived macrophages. Monocyte-derived macrophages isolated from patients with CD were cultured under basal conditions (FBS), stimulated with lipopolysaccharide (LPS), or treated with the selective histone demethylase inhibitor GSK-J4 (30µM) in the presence of LPS for 24 h. Representative Western blot analysis demonstrate changes in protein expression of WNK1, TAK1, phospho-STAT3 (p-STAT3), NF-κB p65, NF-κB B1/B2, and p44 MAPK (ERK) under the indicated treatment condition (**A**). Densitometric quantification of WNK1 (**B**), TAK1 (**C**), pSTAT3 (**D**), NF-κB p65 (**E**), and p44 MAPK (pMAPK) (**F**) is shown, with protein expression normalised to GAPDH. Quantitative RT-PCR analysis further demonstrates modulation of downstream inflammatory signalling components, including STAT3 (**G**), NFKB1 (**H**), NFKB2 (**I**), and NFKB p65 (**J**), following treatment. ∆Ct values were calculated as expression normalised to GAPDH, ∆∆Ct values are expressed as fold change relative to basal conditions. Data are presented as mean ± SEM from CD-derived macrophages (*n* = 9). Statistical significance was determined using appropriate multi-group comparisons; *p* ≤ 0.05 was considered significant

To determine whether the amelioration of cytokine release by GSK-J4 was mediated through the WNK1 and TAK1 signalling pathway via NF-κB and MAPK phosphorylation, CD-derived macrophage protein expression was assessed following LPS stimulation, with and without GSK-J4 treatment 24 h (Fig. 6).

WNK1 expression was significantly upregulated in LPS + GSK-J4-treated cells, while TAK1 expression was downregulated (*p* < 0.05 and *p* < 0.01 respectively) (Fig. 6A-C). Given the role of NF-κB, MAPK and STAT3 signalling pathways in regulating LPS-induced inflammatory cytokines, we further examined the expression of phosphorylated active forms of these signalling molecules.

pSTAT3 (83 kDa) was significantly upregulated following GSK-J4 treatment, both with and without LPS (*p* < 0.01 and *p* < 0.001, respectively) (Fig. 6A and D). The pro-inflammatory mediator NF-κB (65 kDa) exhibited a slight upregulation following LPS treatment, in comparison to LPS + GSK-J4, this was not statistically significant (*p* = 0.15) (Fig. 6A and E). MAPK phosphorylation was significantly upregulated with LPS treatment (*p* < 0.05) and reduced upon LPS + GSK-J4 treatment; however, this reduction was not statistically significant (*p* = 0.27) (Fig. 6A and F).

Despite these changes at protein level, STAT3 RNA expression remained unchanged across treatments (Fig. 6G). Similar expression profile was observed for RNA expression of NF-κB1 (Fig. 6H). However, RNA expression levels of NF-κB B2 and p65 mirrored protein expression trends, with increased expression following LPS treatment and reduced expression upon GSK-J4 treatment (Fig. 6I and J).

## Discussion

**Fig. 7:**
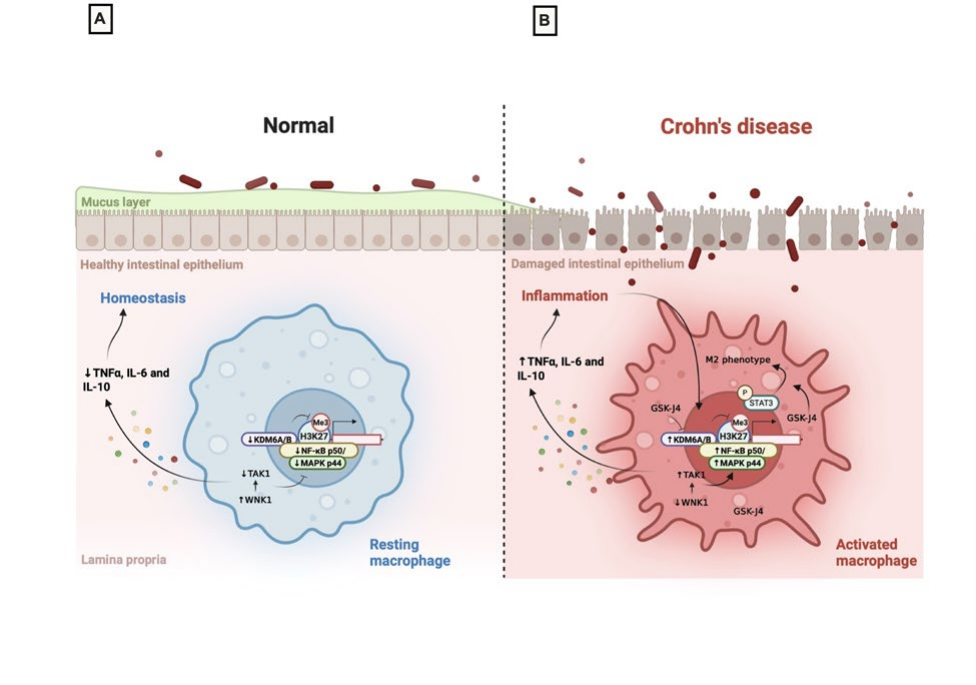
In healthy intestinal macrophages (**A**), low KDM6A/B expression maintains H3K27 trimethylation (H3K27me3), suppressing NF-κB/MAPK activation and limiting cytokine release. In Crohn’s disease (**B**), KDM6A/B are upregulated, H3K27me3 is reduced, and pro-inflammatory cytokines (TNFα, IL-6, IL-10) are elevated, driving epithelial damage. GSK-J4 (**B**), a selective KDM6A/B inhibitor, restores H3K27me3, attenuates cytokine production, and modulates WNK1-TAK1 signalling, representing a novel epigenetic therapeutic strategy for Crohn’s disease treatment

This study investigated the role of H3K27me3 in the pathophysiology of CD and assessed the therapeutic potential of inhibiting its demethylases, KDM6A and KDM6B. Our findings indicate that H3K27me3 expression is significantly reduced in inflamed CD bowel tissue, while KDM6A and KDM6B are upregulated at both the protein and mRNA levels. Similarly, CD-derived macrophages exhibit lower H3K27me3 and increased KDM6A and KDM6B expression compared to macrophages derived from healthy controls (Fig. 7A and B). Treatment with GSK-J4 effectively attenuates this dysregulated expression, increasing H3K27me3 levels while reducing KDM6A and KDM6B expression in an LPS-induced in vitro model (Fig. 7B). Furthermore, GSK-J4 treatment significantly ameliorates the release of inflammatory cytokines IL-6, IL-10 and TNF-α from CD-derived macrophages. Mechanistically, our findings suggest that GSK-J4 can potentially modulates inflammatory cytokine release in association with changes in the WNK1-TAK1 signalling axis, with downstream effects on STAT3 pathways, reinforcing its potential role as an epigenetic modulator in inflammation (Fig. 7B). These findings highlight H3K27me3 regulation as a potential therapeutic target in CD, with GSK-J4 emerging as a promising candidate for modulating disease-associated inflammation (Fig. 7).

H3K27me3 has previously been implicated in inflammatory conditions and its downregulation in CD aligns with findings in dextran sodium sulphate induced colitis mice models [19–21]. In the same model, KDM6B was upregulated while H3K27me3 was downregulated, whereas KDM6A expression was decreased [20]. Similarly, in necrotising enterocolitis, KDM6B expression was increased with a corresponding reduction in H3K27me3 in intestinal tissue from affected infants, compared to those with congenital intestinal malformation [21]. H3K27me3 has been suggested to regulate cell proliferation and inflammation in intestinal epithelial cells via the STAT3 or p38 MAPK pathway [22]. Additionally, H3K27 acetylation has also been implicated in IBD [23]. Our results align with those previous findings and, for the first time, demonstrate dysregulation of H3K27me3 in CD patient samples.

The WNK1-TAK1 signalling pathway is a key regulator of inflammatory responses, particularly in LPS-induced cytokine production, where TAK1 activation facilitates NF-κB nuclear translocation and MAPK phosphorylation, promoting pro-inflammatory gene transcription [17, 18]. TAK1 activation has been observed in both murine models of IBD, and in CD and ulcerative colitis (UC) patient tissues, underscoring its role in chronic intestinal inflammation [18]. Although WNK1 is not currently recognised as a core pathway in CD pathogenesis, in this study, we found that GSK-J4 treatment significantly increased WNK1 expression in LPS-stimulated CD-derived macrophages, while simultaneously reducing TAK1, NF-κB and phosphorylated MAPK expression compared to LPS alone. Given that WNK1 has been shown to suppress TAK1 activation, these findings suggest that modulation of the WNK1-TAK1 axis may contribute to the anti-inflammatory effect of GSK-J4 in CD macrophages. This is further supported by previous studies demonstrating that NF-κB regulates KDM6B expression [10], and that STAT3 and pSTAT3 are elevated in IBD patient T-cells, contributing to inflammatory responses [24, 25]. The observed downregulation of TAK1 following GSK-J4 treatment aligns with prior findings where GSK-J4 suppressed NF-κB and JAK/STAT pathways in murine colitis and necrotising enterocolitis models [21, 26]. Together, these data provide novel insights into the epigenetic regulation of inflammatory signalling in CD, suggesting that GSK-J4-mediated KDM6A/B inhibition can restore H3K27me3 levels, suppress pro-inflammatory mediators, and ultimately disrupt key inflammatory cascades involved in CD pathogenesis. These findings further support the therapeutic potential of GSK-J4 in CD, reinforcing its role as a promising candidate for targeting epigenetic and inflammatory mechanisms in disease modulation. Importantly, we do not propose WNK1 as a disease-initiating pathway in CD, but rather as a regulatory node influencing inflammatory and epigenetic signalling in macrophages within established disease.

Interestingly, we observed a more pronounced LPS-induced cytokine responses in HC-derived macrophages compared to CD-derived macrophages. This may reflect disease-associated macrophage re-programming, whereby chronic exposure to luminal microbial and inflammatory stimuli leads to altered responsiveness to secondary activation signals. In CD, macrophages are persistently exposed to bacterial receptor stimulation [27, 28]. In addition, intestinal macrophages in CD exhibit a transcriptionally and metabolically reprogrammed state [29, 30]. Dysregulation of innate immune pathways, including NOD2 and autophagy-related signalling, further contributes to alter macrophage function and impair microbial clearance [13, 31, 32]. Together, these factors may explain the attenuated cytokine response observed in CD-derived macrophages relative to healthy controls. In addition, assessment of IL-6 receptor expression provides insight into macrophages responsiveness to IL-6 signalling as IL-6R expression determines cellular capacity for classical IL-6 signalling and is dynamically regulated during inflammatory responses [33–36].

The temporal dynamic of IL-10 production should also be considered when interpreting these findings. IL-10 is a key anti-inflammatory cytokine typically induced as a delayed negative feedback response following sustained inflammatory stimulation. In macrophages, LPS-induced IL-10 expression occurs through sequential signalling pathway, with early MyD88-dependent mechanisms followed by later type I interferon-dependent regulation, resulting in peak production at later timepoints such as 24 h [37–39]. This delayed induction plays a central role in limiting excessive inflammation and establishing endotoxin tolerance [40, 41]. Therefore, the increased IL-10 observed at 24 h in our study is consistent with its role as a regulatory cytokine produced in response to sustained LPS stimulation, rather than an indicator of classical M2 polarisation.

Interpretation of these findings should also consider the complexity of macrophage polarisation in vivo, which represents a spectrum of activation states rather than a strict M1/M2 dichotomy [29, 42]. In this study, monocytes were differentiated using M-CSF to generate unpolarised macrophages, which were subsequently stimulated with LPS to induce a pro-inflammatory phenotype. While classical M1 polarisation is often defined by combined interferon-gamma (INF-γ) and LPS stimulation [43, 44], LPS alone is widely used to model inflammatory macrophage activation in vitro [44]. Importantly, markers such as CD68 and CD11c are not exclusively restricted to specific polarisation state [45–47]. In CD, inflamed mucosa is enriched with recruited monocyte-derived inflammatory macrophages characterised by heightened cytokine production, rather than M1/M2 subset [42, 48]. Future studies incorporating additional phenotypic markers such as CD163 and CD206 may further refine macrophage subset characterisation [49, 50]; however, the primary objective of this study was to investigate epigenetic regulation of inflammatory signalling rather than comprehensive macrophage phenotyping.

A key strength of this study is the use of prospectively collected patient samples from individuals with active CD, including those requiring surgical resection due to disease complications or failure of medical therapy. As such, our cohort represents a patient population with severe disease, highlighting the need for alternative therapeutic strategies. Furthermore, our use of full-thickness intestinal resection specimens allows assessment of transmural inflammation, which is a hallmark of CD. Studies relying on endoscopic biopsies alone may not capture the full extent of disease involvement. While murine models are widely used in CD research, they have significant limitations including, species-specific differences in the immune response, lack of genetic diversity, and failure to account for environmental influences [51].

However, our study is limited by its ex vivo and in vitro nature, meaning that the observed effects of GSK-J4 may not fully translate to clinical efficacy. Additionally, patients undergoing surgery for CD have a history of prior therapies, such as immunosuppressive therapy, which may have influenced inflammatory responses and the effect of GSK-J4.

Long-term management of CD requires immunomodulating therapies to control inflammation and prevent complications. Current treatments, including steroids, purine antagonists, antimetabolites, and biologics, have limitations including immunosuppression, infection risk, and malignancy concerns [52, 53]. Moreover, up to one third of CD patients are primary non-responders to anti-TNF therapy, while 23–63% experience secondary loss of response [4, 6, 54]. Given these challenges, novel therapeutic approaches targeting epigenetic regulators such as KDM6A and KDM6B may offer an alternative strategy.

GSK-J4 has shown protection in sepsis models [55] and is being investigated as an anticancer agent [56, 57]. Encouragingly, murine studies have not reported significant toxicity, though the role of KDM6A in embryonic development may limit its use in paediatric populations [58]. Further research should focus on preclinical pharmacokinetic and pharmacodynamic studies, toxicity profiling, and off-target effects, ultimately paving the way for Phase 1 clinical trials.

In conclusion, this study demonstrates for the first time that KDM6A and KDM6B are upregulated in CD, leading to H3K27me3 demethylation and activation of pro-inflammatory pathways. GSK-J4 effectively reverses these epigenetic changes, reducing inflammatory mediators in CD macrophages. Furthermore, GSK-J4 modulates the WNK1-TAK1 signalling pathway, resulting in significant upregulation of WNK1 and STAT3, alongside significant downregulation of TAK1. While no statistically significant suppression of NF-κB/MAPK signalling was observed in this study, these results indicate that GSK-J4 influences key components of inflammatory signalling in CD-derived macrophages. These findings highlight KDM6A and KDM6B as promising therapeutic targets and support further investigation of GSK-J4 as a potential treatment for CD.

## Supplementary Information

Below is the link to the electronic supplementary material.

Supplementary Material 1

Supplementary Material 2

Supplementary Material and Methods

Supplementary Material and Methods PDF

## Data Availability

The datasets generated and/or analysed during the current study are not publicly available due to patient privacy and ethical restrictions. However, de-identified data are available from the corresponding author on reasonable request. All experimental protocols and additional methodological details are provided in the online supplementary materials.
