# Supplementary material for: Histone H3K27 demethylation drives Crohn’s disease inflammation: GSK-J4 as a potential epigenetic therapy
Source: Clin Epigenetics. 2026 May 21;18:144. doi: 10.1186/s13148-026-02165-2 (PMC13371444; doi:10.1186/s13148-026-02165-2)
Supplement: Supplementary file 1 — Supplementary Material 1 [file 13148_2026_2165_MOESM1_ESM.docx]

**Online supplementary material and methods**

**Participants baseline characteristics**

A total of 19 patients suffering from CD and 17 age-matched healthy controls participated in this study. Among the CD patients, 2 provided endoscopic specimens, while 17 provided surgical specimens. Healthy control participants provided blood samples for comparison. The baseline characteristics of all participants are summarised in Table 1

| Supplement table 1. Participants characteristics | | | |
| --- | --- | --- | --- |
| A total of 19 patients suffering from CD and 17 age-matched healthy controls participated in this study. Among the CD patients, 2 provided endoscopic specimens, while 17 provided surgical specimens. Healthy control participants provided blood samples for comparison. The baseline characteristics of all participants are summarised in Table 1. Table 1. Participants characteristics | | | |
|  | **Healthy Controls (n=17)** | **Crohn’s Disease (n=19)** | **P-value** |
| Age in years: mean (SD) | 32 (11) | 32 (11) | 0.87 |
| Gender   - Male, n (%) - Female, n (%) | 8 (47)  9 (53) | 11 (58)  8 (42) | 0.73 |
| BMI kg/m^2^: mean (SD) | 25 (4) | 23 (4) | 0.2 |
| Montreal Classification:   - A1, n (%) - A2, n (%) - L1, n (%) - L2, n (%) - L3, n (%) - B1, n (%) - B2, n (%) - B3, n (%) - Perianal, n (%) |  | 5 (26)  14 (74)  6 (26)  6 (26)  7 (37)  5 (26)  8 (42)  6 (32)  5 (26) |  |
| HBI: mean (SD) |  | 7 (4) |  |
| SES-CD: mean (SD) |  | 5.8 (2) |  |
| Immunosuppressive therapy, n (%) |  | 12 (63) |  |
| Active disease on final histology, n (%) |  | 11 (58) |  |
| Anti-TNF, n (%) |  | 6 (32) |  |
| Anti-IL12/IL23, n (%) |  | 3 (16) |  |
| WCC, x10^9^/L: mean (SD) |  | 8.6 (3) |  |
| Hb, g/dL: mean (SD) |  | 12.7 (1.7) |  |
| CRP, mg/L: median (range) |  | 9 (0.6-110) |  |
| BMI, Body Mass Index; CRP, C-Reactive Protein; Hb, Haemoglobin; HBI, Harvey-Bradshaw Index; SES-CD, Simplified Endoscopic Score for Crohn’s Disease; TNF, Tumour Necrosis Factor; WCC, White Cell Count. | | | |

**Methods:**

To evaluate the percentage of macrophages expressing the demethylases KDM6A and KDM6B, and H3k27me3, immunofluorescence imaging was performed. This technique enabled the assessment of macrophage presence within tissue and their expression of KDM6A and KDM6B.

**Tissue Preparation and Staining**

Colon tissue samples from inflamed and unaffected regions of the same patient were fixed in 10% formalin. Serial sections (10 µm) were prepared for histological analysis. Rehydration was achieved by immersing slides in a graded ethanol series (100% twice, followed by 96%, 90%, 80%, and 70%) for 5 minutes each, with a subsequent 5-minute rinse in deionized water.

Heat -Antigen retrieval was conducted using a pH 6 citrate buffer (1:10 dilution of a stock solution), heated at100^o^C for 10 minutes at 20-second intervals. After cooling to room temperature, slides were rinsed twice in Tris-buffered saline with 0.1% Tween (TBST, pH 7.4). Permeabilization was carried out by incubating slides in 0.2% Triton X-100 in phosphate-buffered saline (PBS, pH 7.4) for 10 minutes, followed by two TBST rinses. Hydrophobic barriers were drawn around samples using a Dako barrier pen. Slides were then blocked for 30 minutes at room temperature with 5% donkey serum- blocking solution. Primary antibodies were applied at validated dilution (Table 1) in an antibody dilution buffer. Slides were incubated at 37°C for 1 hour, followed by 2 hours at room temperature, and overnight at 4°C. The next day, slides were rinsed three times with TBST, and appropriate secondary antibodies were applied for 30 minutes at room temperature, protected from light. After PBS washes, nuclear counterstaining was performed using DAPI (1:1000 dilution) for 3 minutes. Slides were mounted with coverslips using minimal mounting medium and stored at –20°C prior until imaged.

| Supplement table 2. | | | | |
| --- | --- | --- | --- | --- |
| Target | Primary AB | Final concentration | Secondary AB | Final concentration |
| KDM6A | abcam, ab36938, Rabbit. | 1:300 | Thermo Fishe scientific, A-11034, Goat, Alexa Fluor™ 488. | 1:250 |
| KDM6B | NOVUS a biotechen brand, NBP1-06640AF488, Rabbit, Conjugate Alexa Fluor 488. | | | 1:250 |
| CD11C | Abcam, ab23602, Mouse | 1:300 | Thermo Fishe scientific, R-6393, Goat, Rhodamine Red^TM^-X . | 1:250 |
| CD68 | abcam, ab955, Mouse. | 1:300 | Thermo Fishe scientific, R-6393, Goat, Rhodamine Red^TM^-X. | 1:250 |
| Tri-Methyl-Histone H3 | Cell Signaling, 9733, Rabbit | 1:300 | Thermo Fishe scientific, A-11034, Goat, Alexa Fluor™ 488. | 1:250 |

**Imaging and Analysis**

Images were captured using an Axioscan microscope. Regions of interest (three per sample) were cropped and analyzed using FIJI (ImageJ) software (version 2.15.1).

**Immuno-cytology**

Cells were first were seeded into 8-chambered slides (BD Falcon, Oxford, UK) and grown in growth medium containing M-CSF. After reaching the required confluency, the cells were treated with 30 μM of GSKJ4 with or without E. coli Lipopolysaccharide (LPS) (10 ng/ml) for 24 hrs before terminating the experiment. For staining, cells were fixed with 4% paraformaldehyde (PFA; Sigma-Aldrich, Dorset, UK), followed by permeabilization in 0.1% Triton X-100 (Sigma-Aldrich, Dorset, UK) for 10 minutes. After aspiration of supernatants, cells were washed thrice with phosphate-buffered saline (PBS), followed by incubation at RT for 10 minutes in blocking solution comprising of 3% bovine serum albumin (BSA) in 0.01% Triton X-100. Both primary and secondary antibodies were diluted in the blocking solution. Cells were incubated with primary antibody overnight at 4°C followed by washes to remove non-specific binding. The appropriate secondary antibody incubation was performed for one hour (h) at RT before addition of the fluorescent nuclear stain, DAPI (Vector Laboratories, Southgate UK).

The following primary antibodies were used: Rabbit monoclonal anti-trimethyl histone H3 (Cell Signalling Technology, Hitchin, UK), rabbit polyclonal anti-KDM6A (1:500, Abcam, Cambridge, UK), polyclonal rabbit anti-KDM6B (1:500, Abcam, Cambridge, UK). Alexafluor-488 donkey anti-rabbit IgG (1:1000, A21206; Invitrogen, Paisley, UK) was used as a secondary for trimethyl histone H3, KDM6A and KDM6B staining. Omission of the primary antibody served as a negative control. Confocal imaging was performed using a Leica TCS SPE upright microscope (Leica Microsystems, Milton Keynes, UK) and Z-stack images were acquired and analysed using proprietary Leica LAS X Software (Leica Microsystems).

**Western blotting**

Snap-frozen and crushed Tissue sections and cells were lysed with RIPA buffer (Sigma-Aldrich, Dorset, UK) containing protease and phosphatase inhibitors. Lysed samples were centrifuged at 16,100g for 15 min at 4°C protein lysate supernatant collected and stored at -80 °C until use. Western blotting analysis was performed on protein samples (10 μg) using NuPAGE MOPS SDS Buffer Kit (Invitrogen, Paisley, UK) following the manufacturer’s guide. Protein bands were visualized using the enhanced chemiluminescence plus reagent detection system (GE Healthcare, Little Chalfont, Buckinghamshire, UK) and imaged via a Gel-Doc system (Syngene, Cambridge, UK). ImageJ (National Institute of Mental Health, Bethesda, MD, USA) was used to compare the density of the bands relative to GAPDH as loading control for primary antibodies (Table- Supp).

**Real-time PCR**

RNA was extracted from homogenized tissue samples or cell pellets using RLT buffer and purified using the RNeasy Mini Kit (Qiagen, Manchester, UK). The extraction process involved chloroform phase separation, isopropanol precipitation, and ethanol washing. The RNA pellet was resuspended in RNase-free water. For qRT-PCR, a one-step kit (Qiagen, Manchester, UK) was used, with reverse transcription at 50°C for 30 minutes, initial denaturation at 95°C for 15 minutes, and 40 cycles of 94°C for 15 seconds and 60°C for 30 seconds. Data analysis was performed to determine relative gene expression levels.

**CELL CULTURE**

Monocytes were enriched from peripheral blood by incubating whole blood with the RosetteSep^TM^ human Monocyte Enrichment Cocktail (STEMCELL Technologies, Cambridge, UK) at room temperature (RT) for 30 minutes. The enriched blood was then diluted with RPMI 1640 (Thermofisher Scientific, UK) and peripheral blood mononuclear cells from the buffy coat separated using a standard technique for blood by layering over Ficoll Paque Plus (Cytiva, Sweden) before centrifugation. Monocyte-derived macrophages were differentiated and cultured in growth medium comprising RPMI 1640 plus l-glutamine (Sigma-Aldrich, Dorset, UK) with 10% foetal bovine serum (FBS; Gibco, UK) and 1% penicillin/streptomycin (Sigma-Aldrich, Dorset, UK) in culture 6-well plates for 7-10 days. Recombinant human macrophage colony-stimulating factor (M-CSF; Peprotech, USA) was added at a concentration of 30 ng/ml. Cell culture medium was replaced every 3 days with fresh growth media containing M-CSF.

**Treatment of macrophages with GSK-J4 compound**

Human blood-derived primary macrophages were cultured with GSKJ4, a dual inhibitor of H3K27me3 demethylases KDM6B and KDM6A at a concentration of 30 μM with or without E. coli LPS (10 ng/ml) for 1, 6 and/or 24 hrs before terminating the experiment with cell-lysis.

**Cytokine Assay**

Human recombinant TNF-alpha, IL-6, IL10, and INF alpha were measured from cell culture supernatants using enzyme-linked immunosorbent assay (ELISA) kits, performed according to the manufacturer’s instructions (R&D Systems, Thermofisher Scientific).

**Cell viability and cytotoxicity assays**

To assess the concentration-dependent effects of GSKJ4 (10 and 30μM), macrophages were seeded onto 96-well (MTS assay) plates at a density of 1×10^4^ cells/mL (total volume 100 μL or 2 mL, respectively). Cells were grown in RPMI basal medium containing 10% FBS (growth medium), and after 24h, the media was replaced with just RPMI basal medium for 48h to growth-arrest cells. Subsequently, the cells were incubated in growth medium for 4 days in the absence and presence of the test agent. Proliferation responses were compared to cells incubated with no added growth factors over the same time period (the time control). Each intervention was performed in quintuplicate (MTS).

Cytotoxicity of the drug doses was assessed using an MTS cell proliferation assay kit (Promega, Southampton, UK), a colorimetric method for determining the number of viable cells based on the cleavage of MTS (3-(4,5-dimethylthiazol-2-yl)-5-(3-carboxymethoxyphenyl)-2-(4-sulfophenyl)-2H-tetrazolium, inner salt) to formazan by cellular mitochondrial dehydrogenases. An increase in cell number leads to a proportional increase in the amount of formazan dye formed, which can be quantified by measuring the absorbance of the dye solution at 490nm using a Versamax Microplate Reader (Sunnyvale, CA, USA). For each drug concentration, absorbance was measured from five wells and the average was taken. The background absorbance was corrected by subtracting the average absorbance from the ‘no cell’ control wells from all other absorbance values.

**Patient and Public Involvement (PPI)**

Patients and members of the public were actively involved at all stages of this study. The original research hypothesis and concept were co-developed with the late Michael Seres, a pioneering patient advocate in Crohn’s disease. Michael reviewed and shaped the early study design and priorities, ensuring alignment with patient needs and translational relevance.

Following Michael’s passing, his son Aaron Seres continued to lead patient and public involvement activities. Under his coordination, a dedicated group of Crohn’s disease patients was engaged to review the full study protocol, including the design, methodology, and burden of participation. This group provided direct feedback on patient-facing documents such as the participant information leaflet and consent forms, resulting in refinements to the language, format, and clarity of these materials to enhance accessibility and comprehension.

The PPI group also contributed feedback on feasibility, acceptability of sampling schedules, and follow-up logistics. Their ongoing input shaped the recruitment process and informed adjustments to optimise participant experience.

Although no formal quantitative assessment of patient burden was performed, qualitative feedback from the group was used to minimise inconvenience and enhance ethical standards. Plans for dissemination of results to participants and for translational development of this research are already in place and will continue to incorporate patient input.
