# Supplementary material for: Histone H3K27 demethylation drives Crohn’s disease inflammation: GSK-J4 as a potential epigenetic therapy
Source: Clin Epigenetics. 2026 May 21;18:144. doi: 10.1186/s13148-026-02165-2 (PMC13371444; doi:10.1186/s13148-026-02165-2)
Supplement: Supplementary file 2 — Supplementary Material 2 [file 13148_2026_2165_MOESM2_ESM.docx]

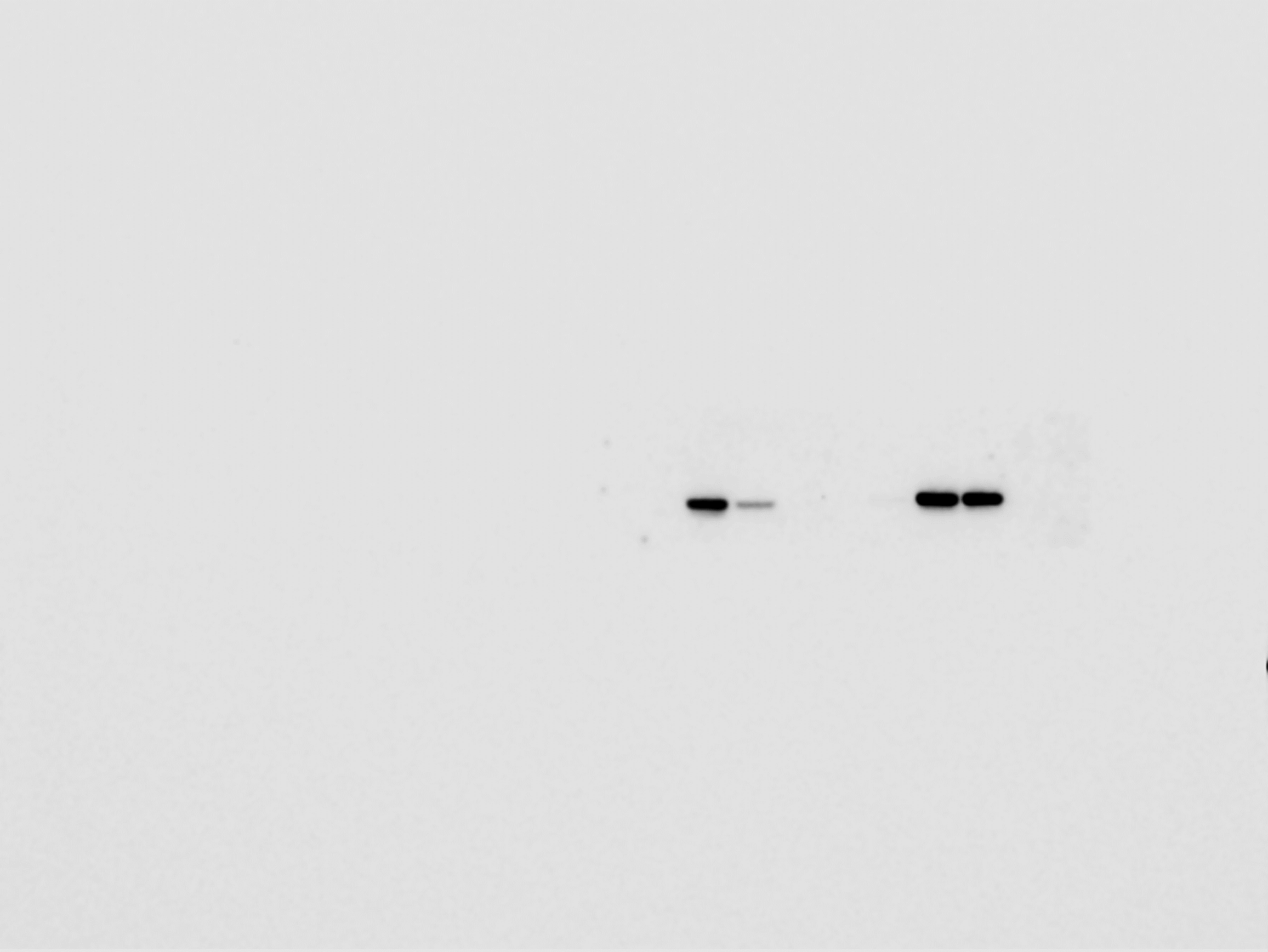

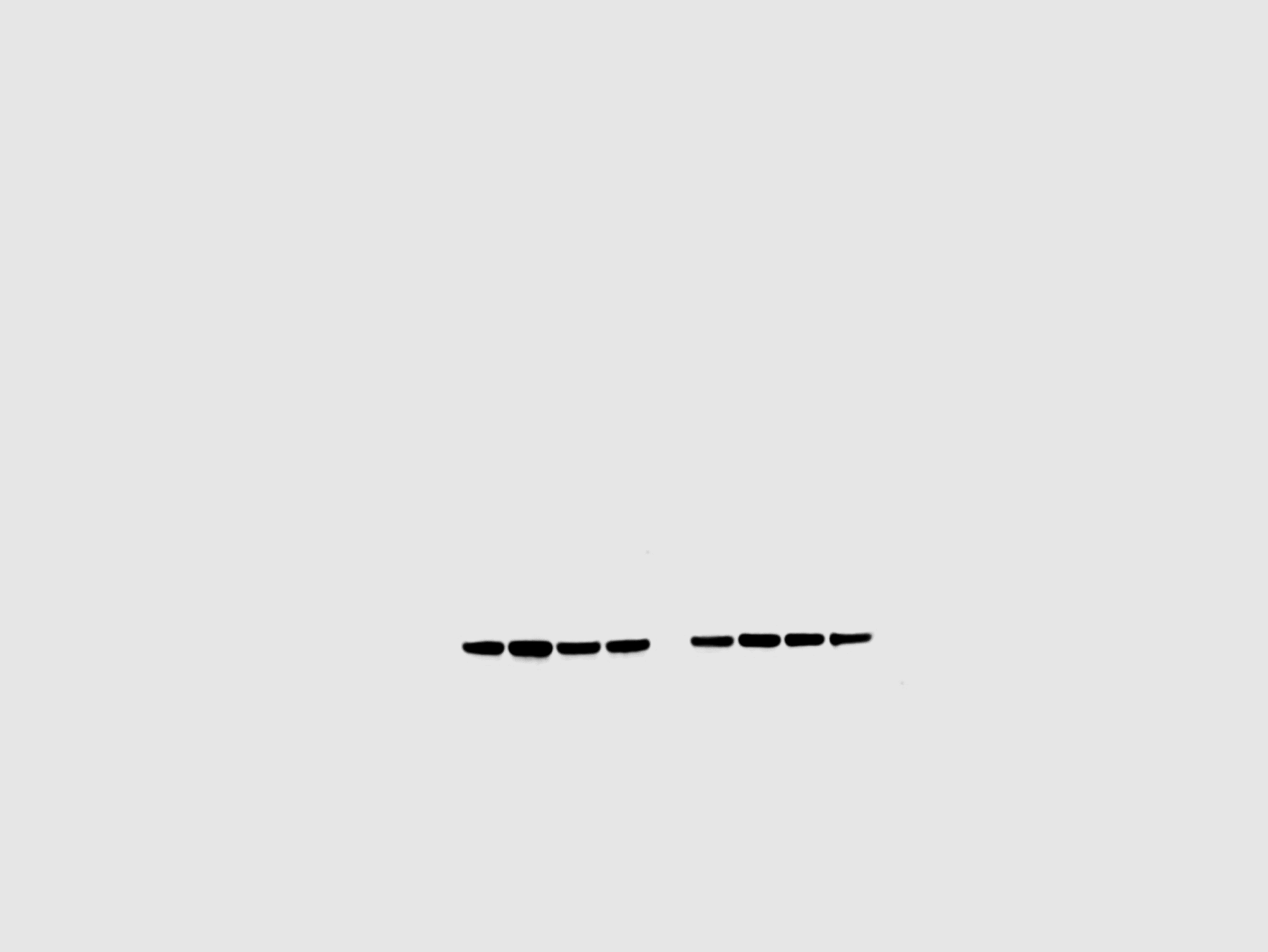


**pSTAT3**

**FBS**

**LPS**

**LPS + GSKJ4 30uM**

**GSKJ4 30uM**

**STAT3**


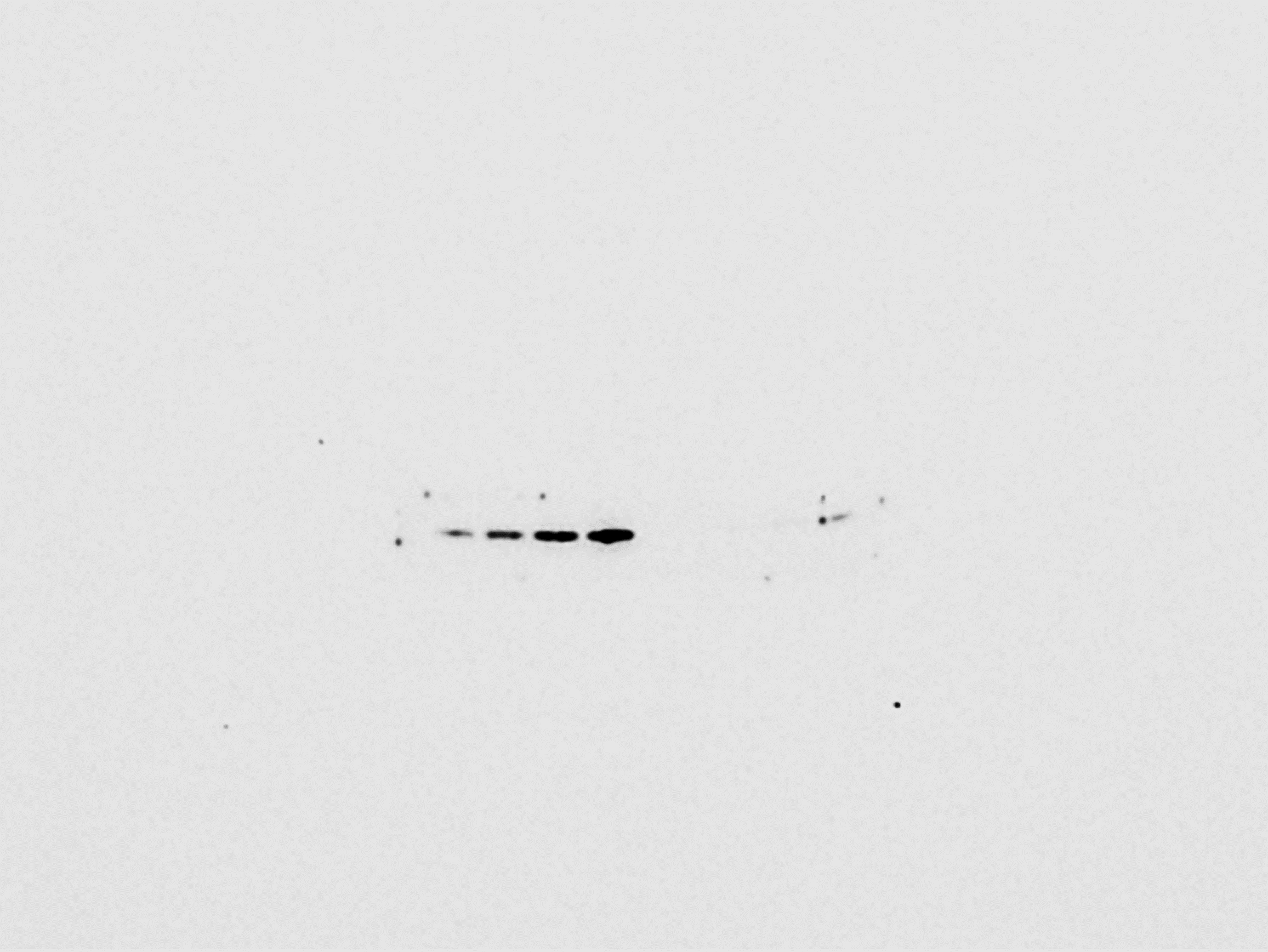


**Tri methyl Histone H3**


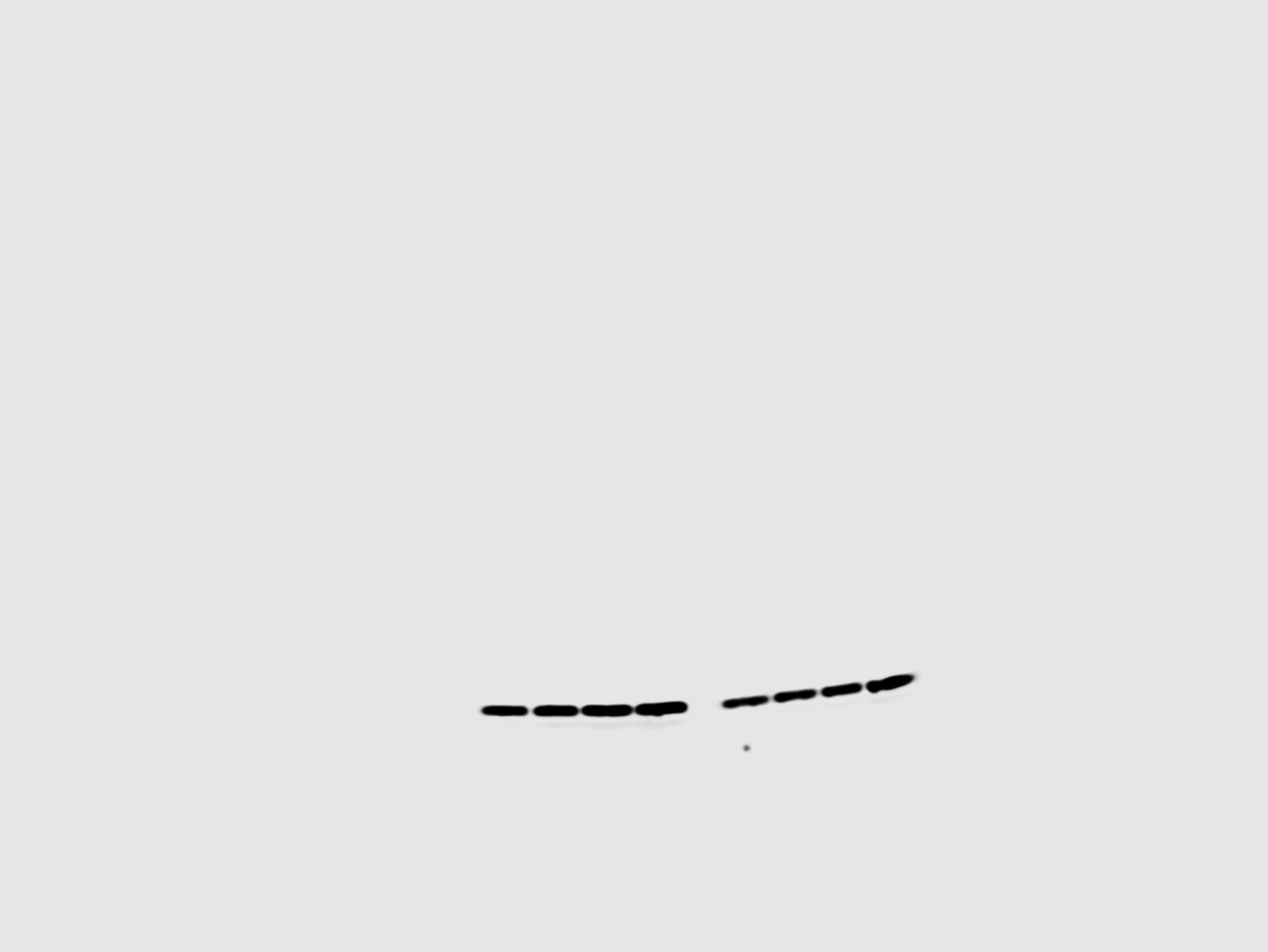


**Histone H3**


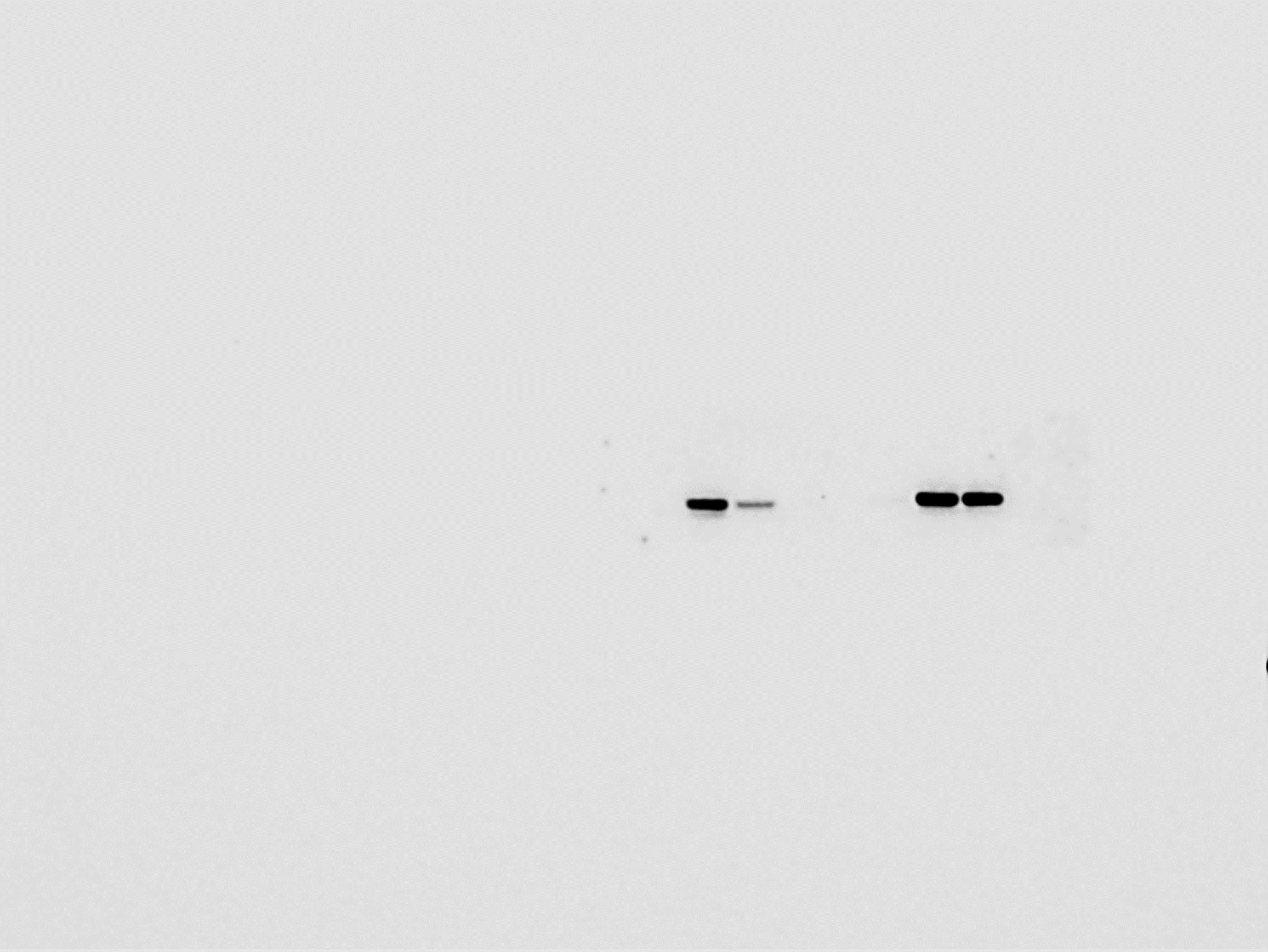


**pSTAT3**


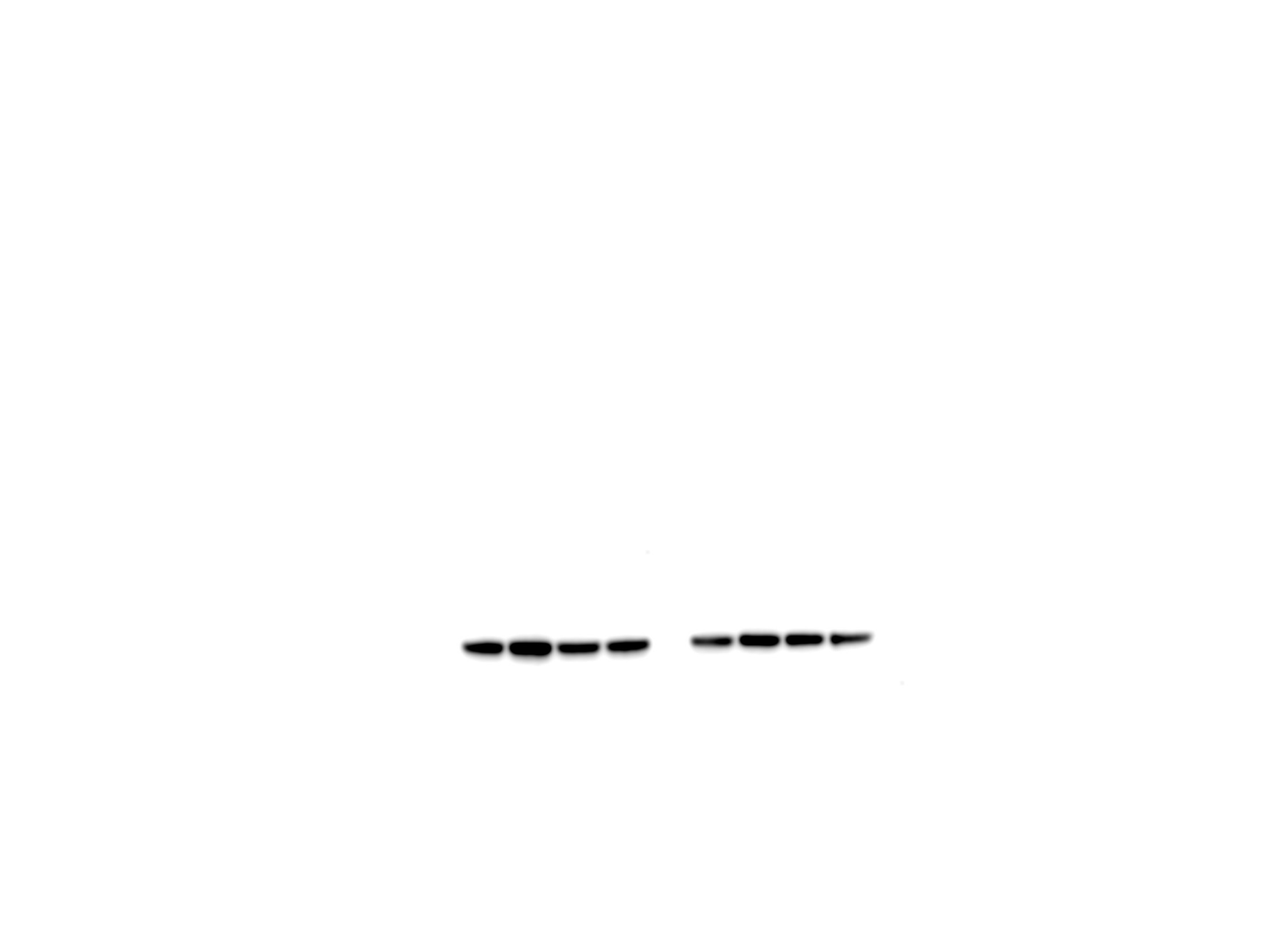


**STAT3**

**Tri methyl Histone H3**


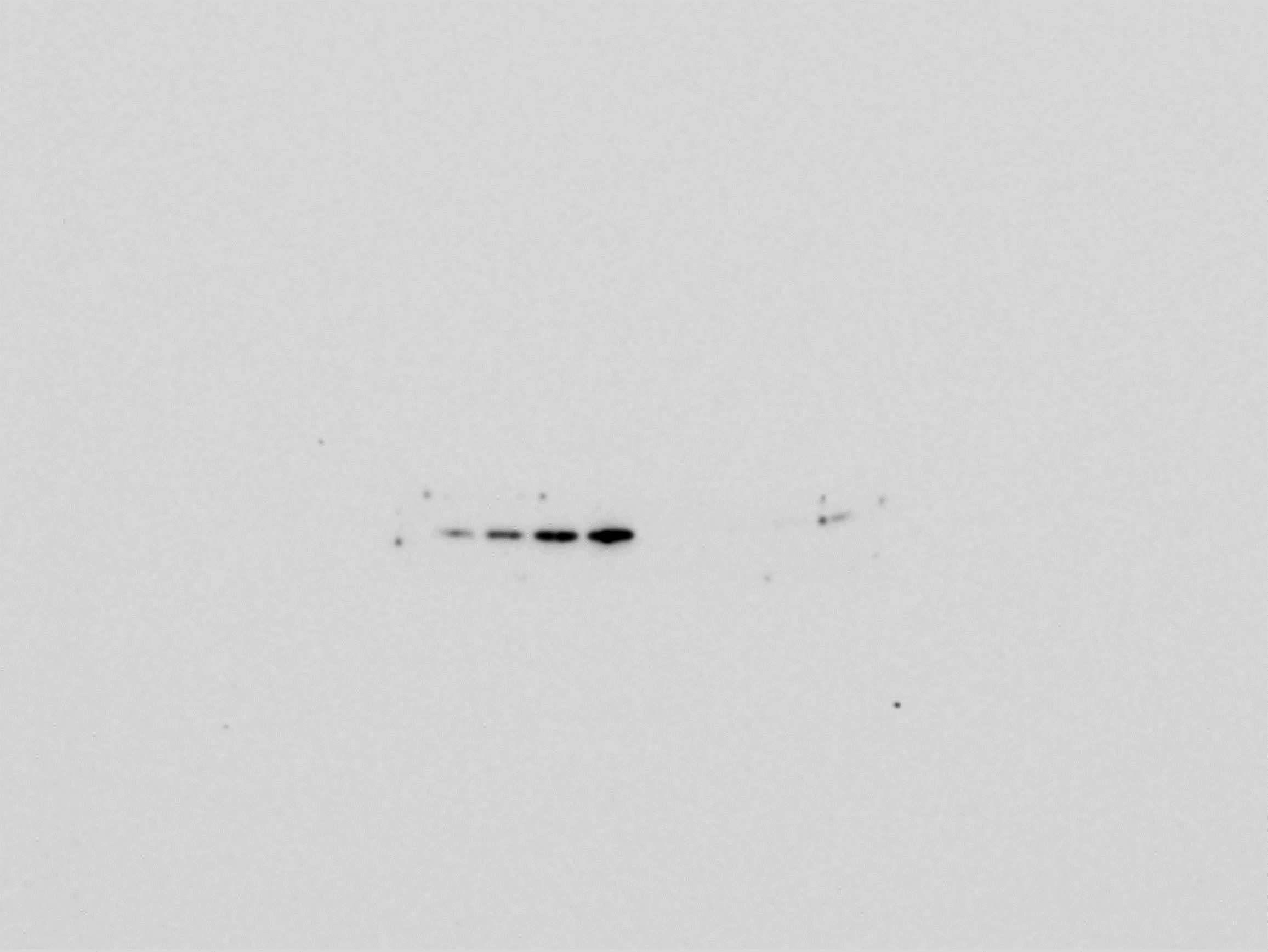


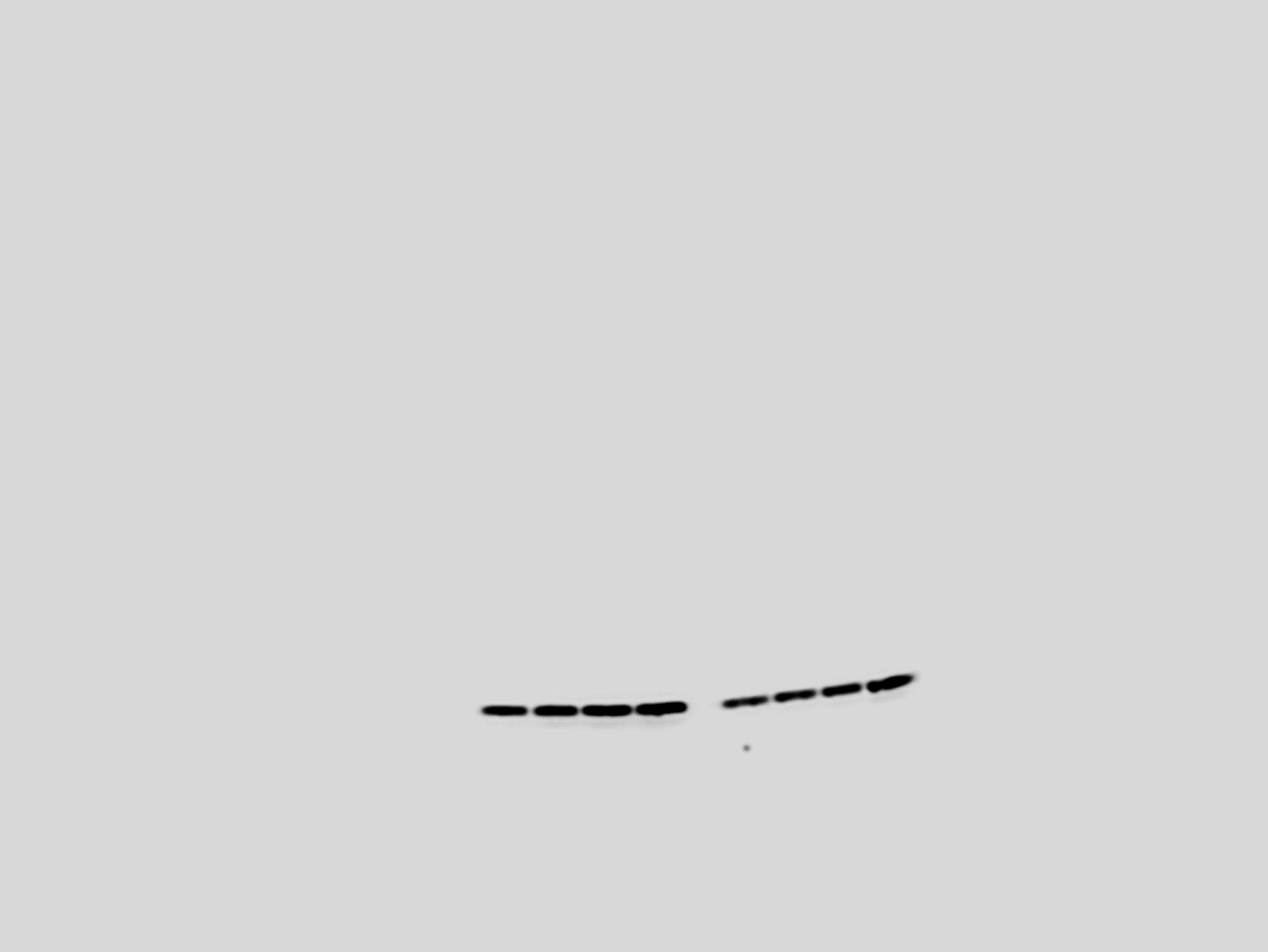


**Histone H3**


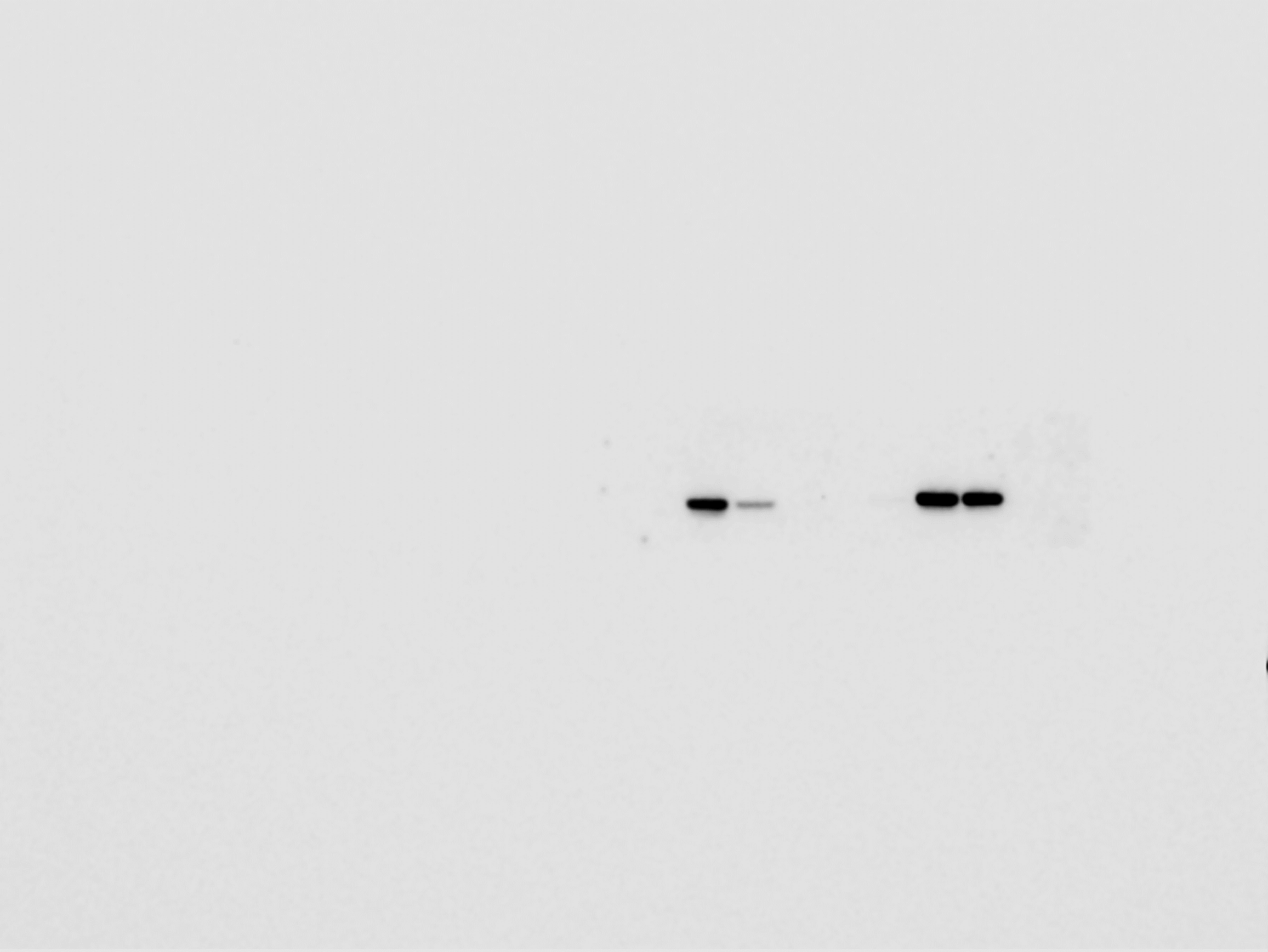


pSTAT3


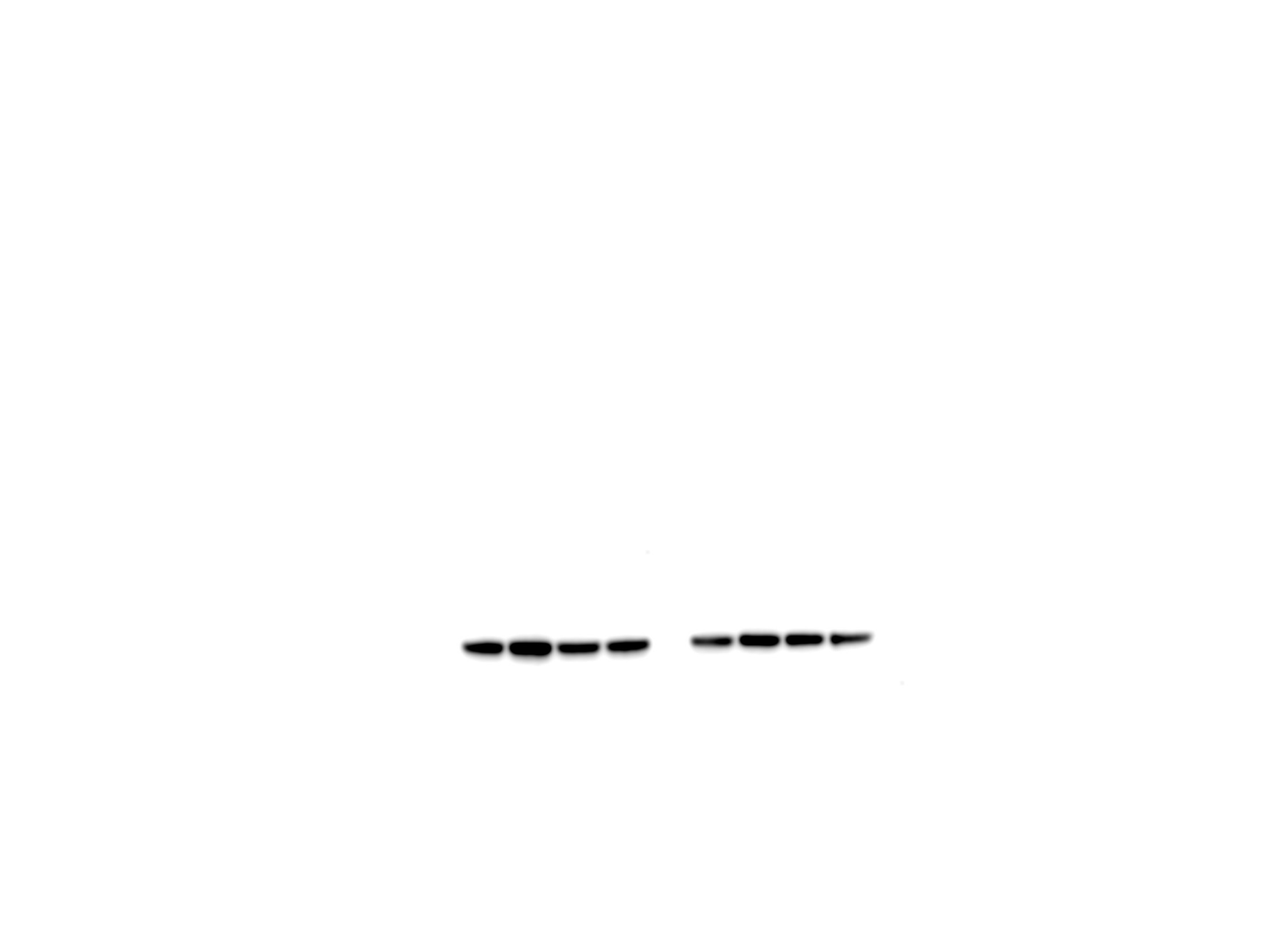


STAT3


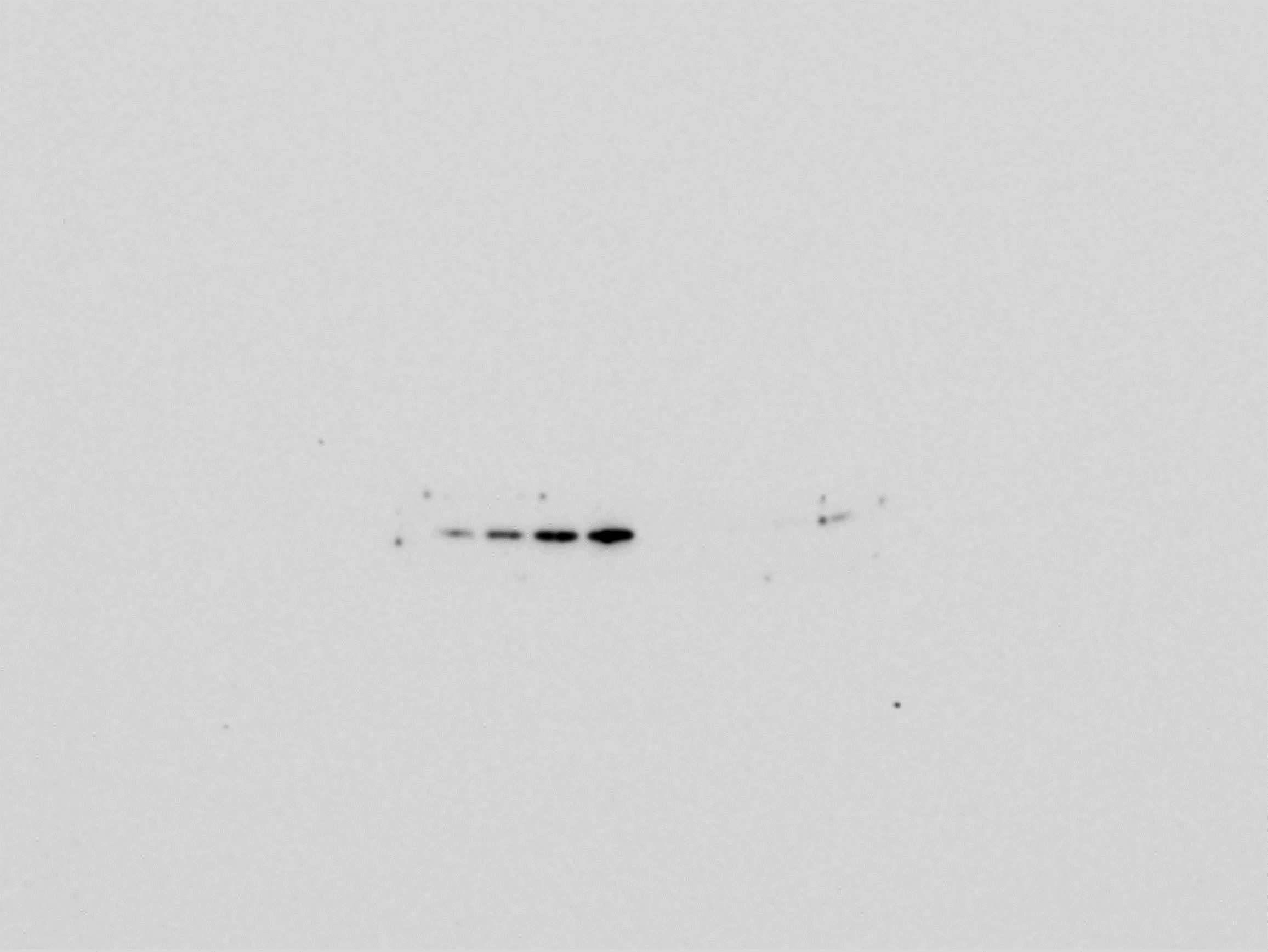

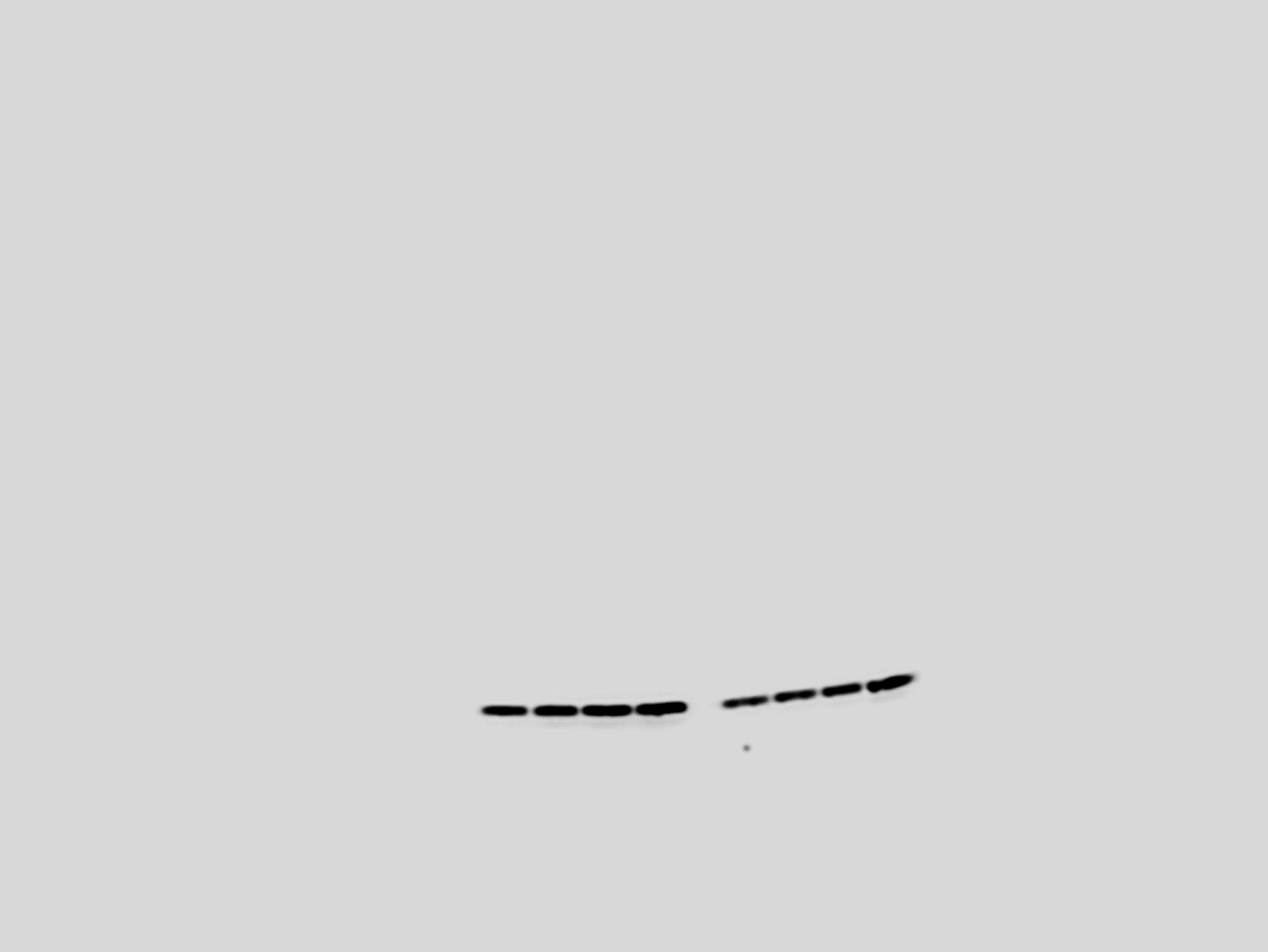


H3K27me3

Histone H3


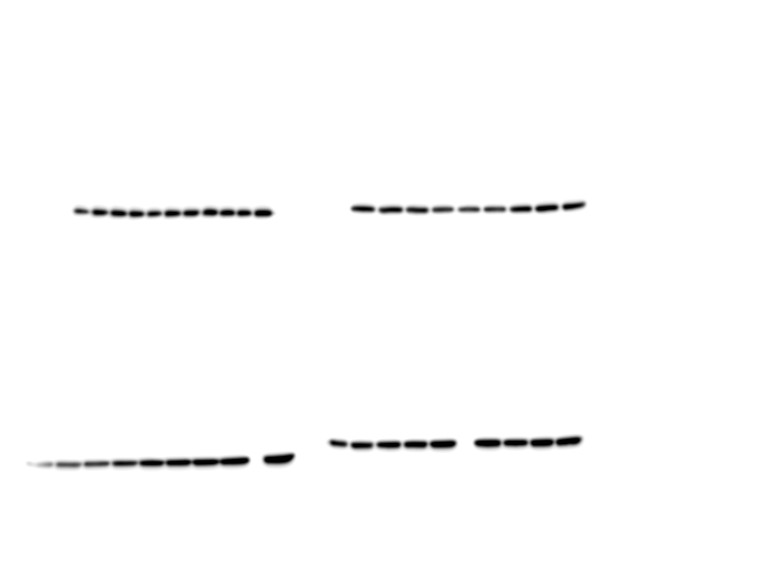


GAPDH


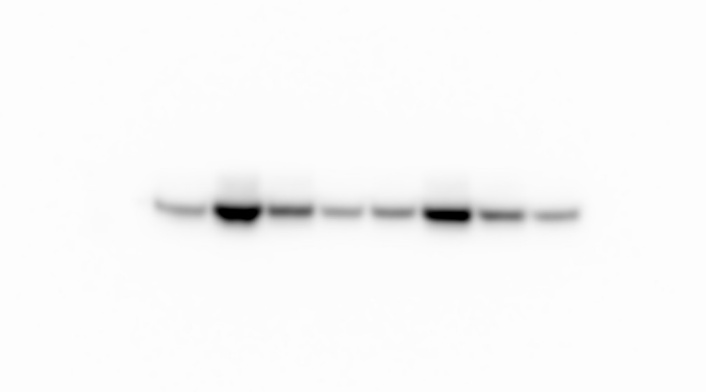


KDM6A


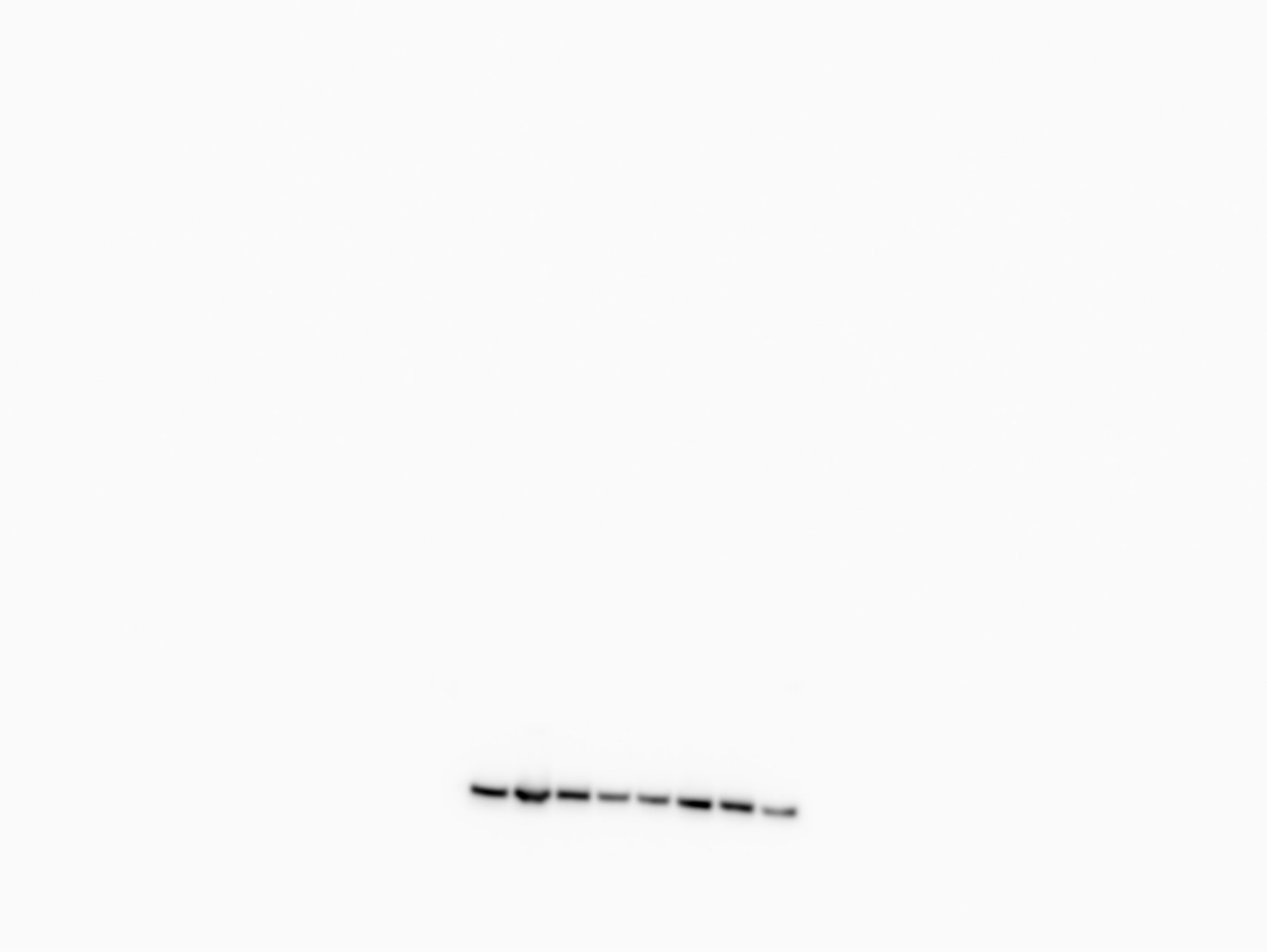


KDM6B


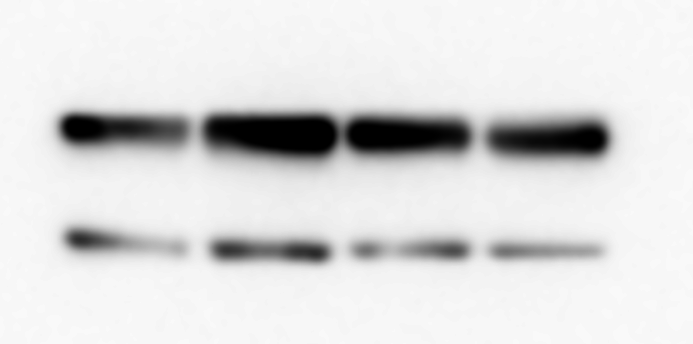

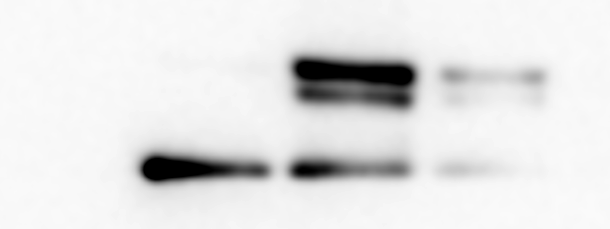

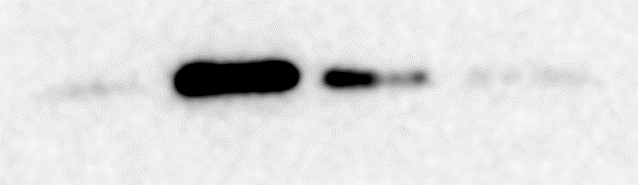

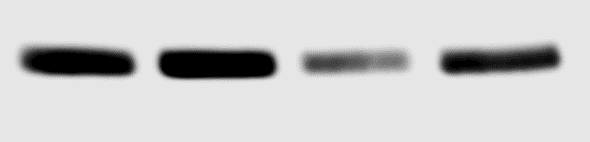

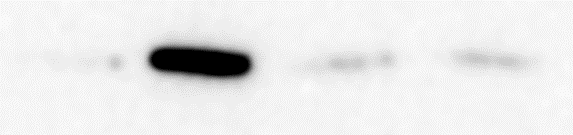

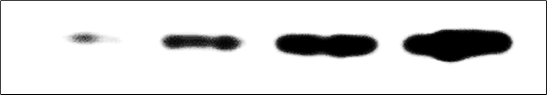

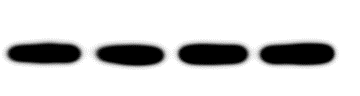


**NFKB p65**

**pSTAT3**

**WNK1**

**TAK1**

**p44 MAPK (ERK)**

**Tri methyl Histone H3**

**FBS**

**LPS**

**LPS + GSKJ4 30uM**

**GSKJ4 30uM**

**GAPDH**

**NFKB p50**

**GSKJ4 30uM**

**LPS + GSKJ4 30uM**

**LPS**

**FBS**


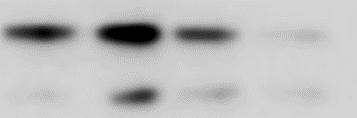

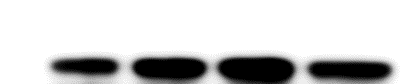

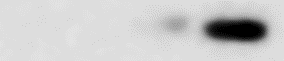


**NFKB p65**

**NFKB p50**

**KDM6A**

**p44 MAPK (ERK)**

**H3K27me3**

**WNK1**

**GAPDH**

**Histone H3**

**pSTAT3**

**GSKJ4 30uM**

**LPS + GSKJ4 30uM**

**LPS**

**FBS**


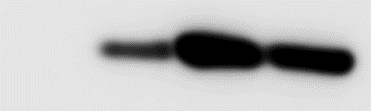

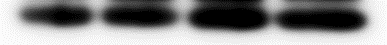

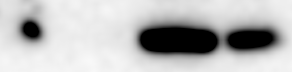


**WNK1**

**H3K27me3**

**GAPDH**

**Histone H3**

**KDM6A**

**pSTAT3**

**NFKB p65**

**NFKB p50**

**p44 MAPK (ERK)**


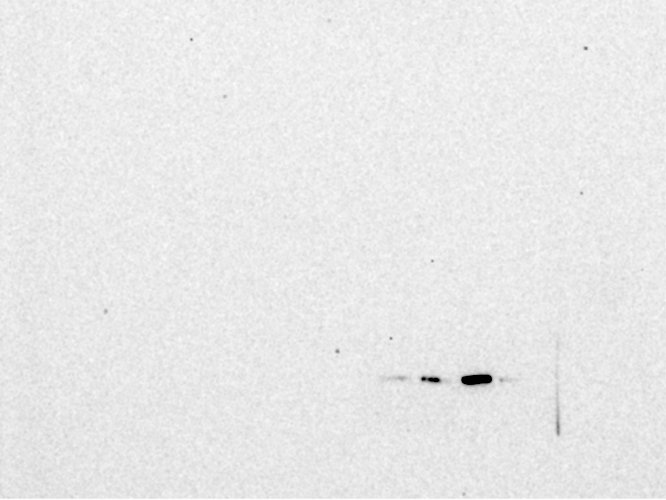

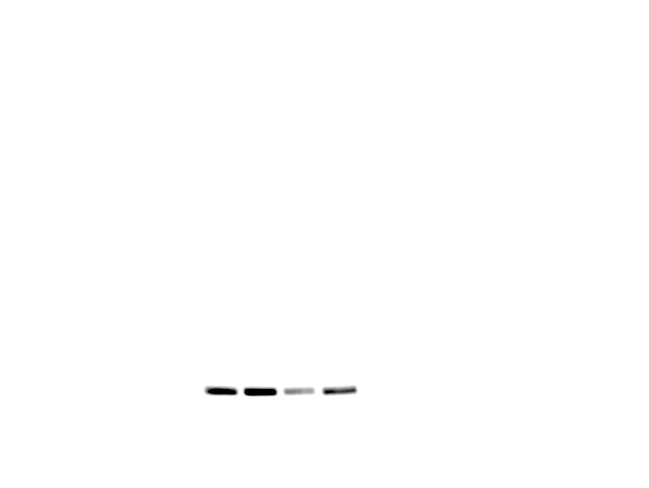

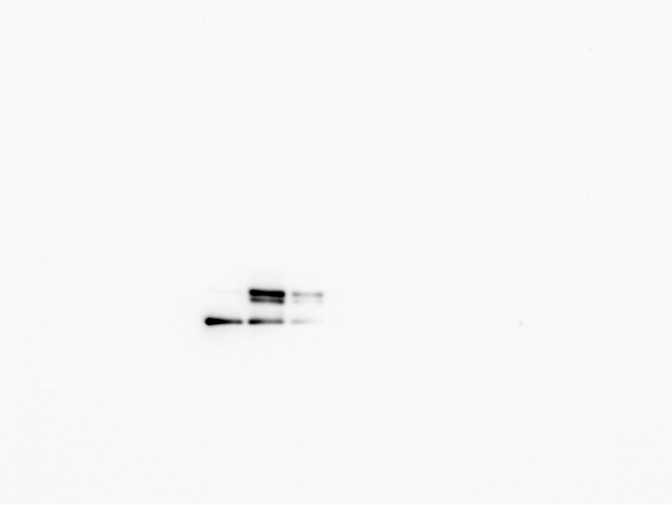

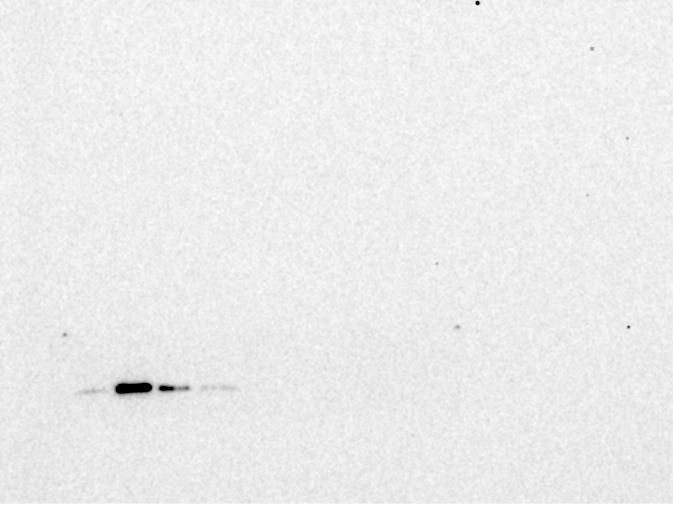

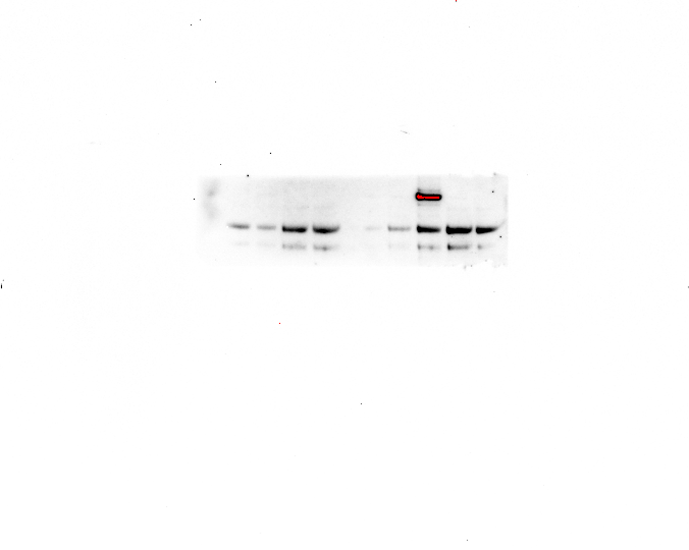

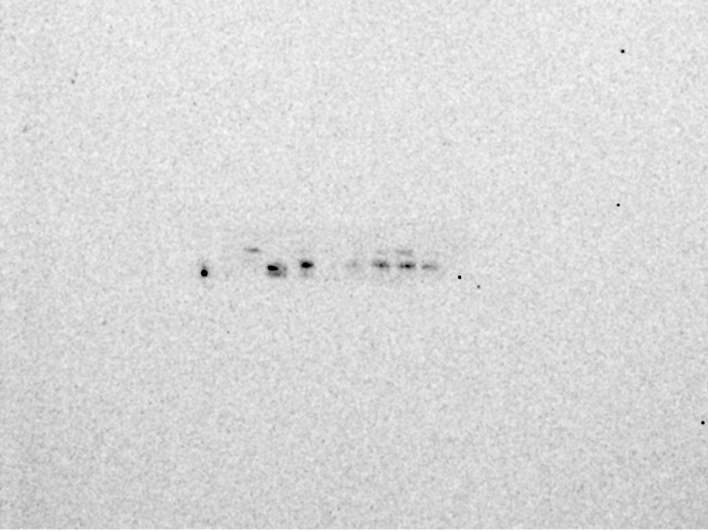

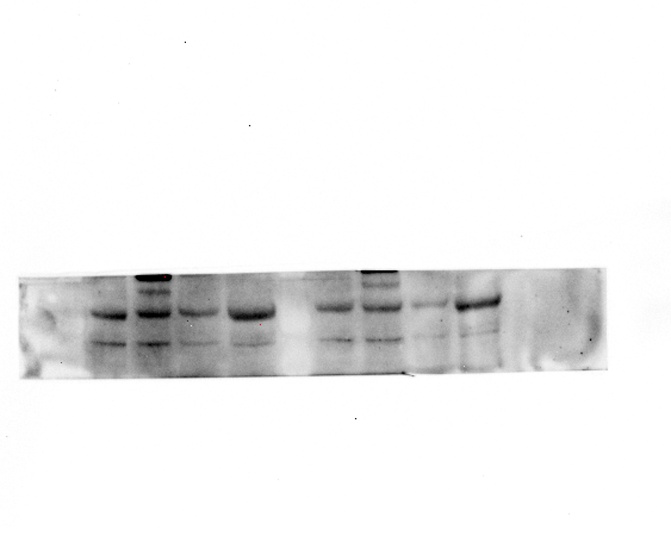


TAK1

WNK1

pSTAT3

MAPK

NFKB

TAK1

WNK1

Tissue extracts

**GAPDH**

**KDM6A**

**Unaffected**

**Affected**


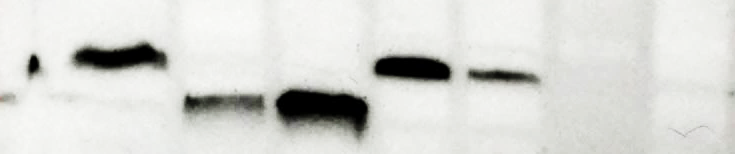


**KDM6B**


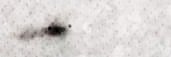


**H3K27me3**

KDM6A


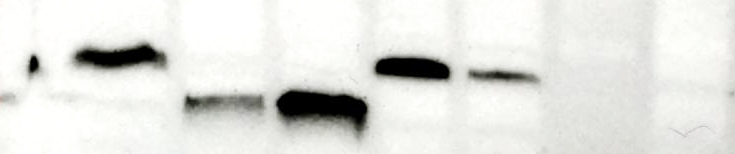


KDM6B


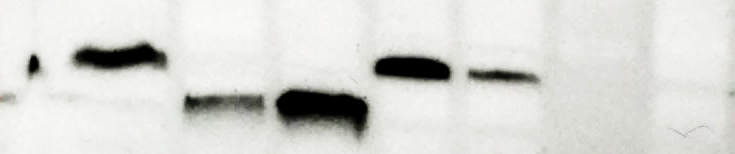


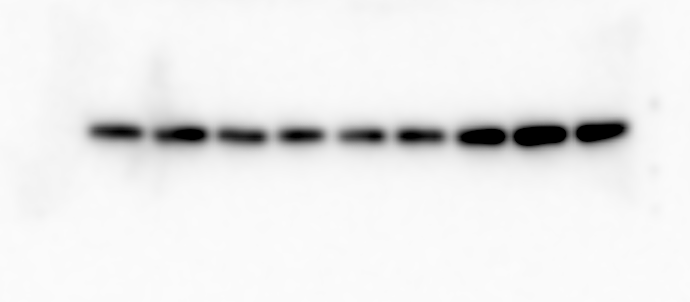


GAPDH


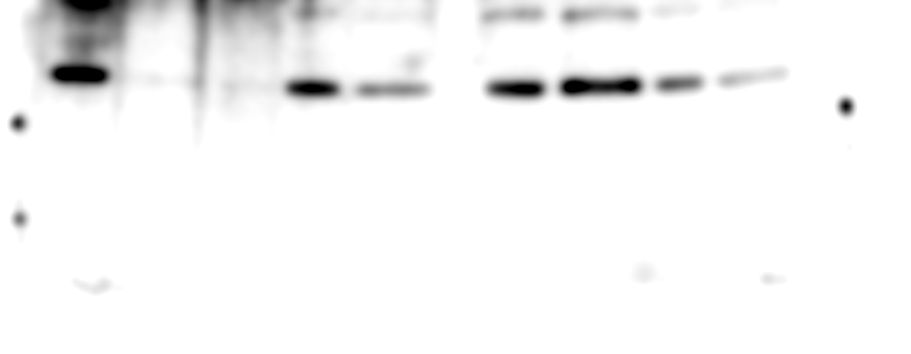


H3K27me3
